# Supplementary material for: GyrI-like proteins catalyze cyclopropanoid hydrolysis to confer cellular protection
Source: Nat Commun. 2017 Nov 14;8:1485. doi: 10.1038/s41467-017-01508-1 (PMC5684135; doi:10.1038/s41467-017-01508-1)
Supplement: Supplementary file 1 — Supplementary Information [file 41467_2017_1508_MOESM1_ESM.pdf]

Supplementary Information

GyrI-like proteins catalyze cyclopropanoid hydrolysis to confer cellular protection

Hua Yuan[1†], Jinru Zhang[1†], Yujuan Cai[1], Sheng Wu[1], Kui Yang[1], H. C. Stephen Chan[2], Wei Huang[1], Wen-Bing Jin[1], Yan Li[1], Yue Yin[1], Yasuhiro Igarashi[3], Shuguang Yuan[4*], Jiahai Zhou[1*], and Gong-Li Tang[1*]

[1]State Key Laboratory of Bio-organic and Natural Products Chemistry, Shanghai Institute of Organic Chemistry, Chinese Academy of Sciences, 345 Lingling Road, Shanghai 200032, China

[2]Faculty of Life Sciences, University of Bradford, Bradford, West Yorkshire, BD7 1DP, United Kingdom

[3]Biotechnology Research Center, Toyama Prefectural University, 5180 Kurokawa, Imizu, Toyama 939-0398, Japan

[4]Laboratory of Physical Chemistry of Polymers and Membranes, Ecole Polytechnique Fédérale de Lausanne (EPFL), CH B3 495 (Bâtiment CH) Station 6, Lausanne CH-1015, Switzerland

[†]These authors contributed equally to this work.

*Correspondence and requests for materials should be addressed to G.-L.T. (email: gltang@sioc.ac.cn), to J. Zhou (email: jiahai@sioc.ac.cn) or to S.Y. (email: shuguang.yuan@gmail.com).

Supplementary Tables and Figures

Supplementary Table 1 | Primers used in this study

Primers	Sequences (5' to 3')	Comments
R7KO1	ccgtcggtctgggaggctgttgccgtttcccttccgatgattccggggatccgtcgacc	To construct the *ΔytkR7* mutant
R7KO2	ccgcagccgctcgggcgcggtgcgctcggggctgtttcatgtaggctggagctgcttc	
R7very1	tcggtctgggaggctgttg	To verify the *ΔytkR7* mutant
R7very2	gcccttcgtcgcatacctg	
R7Re1	acatatgatgcccagtgcagcaaagaaga	To complement the *ΔytkR7* mutant; to overexpress *ytkR7* in *E. coli*
R7Re2	agaattcaagcttactcgagcacctgaacgggctggcgga	
c10R6F	acatatgatgcagaactacgacgtcaag	To complement the *ΔytkR7* mutant; to overexpress *c10R6* in *E. coli*
c10R6R	agaattcaagcttactcgaggacgcccttcggttcgacgggc	
sbmC1	acatatgatgaactacgagattaagcag	For overexpression of *sbmC* in *E. coli*
sbmC2	agaattcaagcttactcgaggtgatgttttggctgcaccgca	
rob1	acatatggatcaggccggcattattcg	For overexpression of *rob* in *E. coli*
rob2	gaagcttactcgagacgacggatcggaatcagca	
8481F	acatatgatgtccctgggcaacaccacgc	For overexpression of *SHJG_8481* in *E. coli*
8481R	agaattcaagcttactcgagccggccccgtcggcgtaccggc	

Supplementary Table 2 | Strains and plasmids used in this study

Strains/ plasmids	Comments	Source/reference
Streptomyces		
sp. TP-A0356	Yatakemycin producer	1
sp. TG1310	A *ytkR7* inactivating mutant	This study
sp. TG1311	*ytkR7* complementary into TG1310	This study
sp. TG1312	*c10R6* complementary into TG1310	This study
zelensis NRRL 11183	CC-1065 producer	2
E. coli		
DH5a	Host used for plasmid construction	Lab. collection
BL21(DE3)	Host used for gene overexpression	Lab. collection
BW25113/pIJ790	Host used for PCR-targeting	3
S17-1	Host used for *E. coli*-*Streptomyces* conjugation	4
Plasmids		
pSET152	Used for complementation in *Streptomyces*	4
pET-28a(+)	Plasmid used for gene overexpression	Lab. collection
pET-37b(+)	Plasmid used for gene overexpression	Lab. collection
pTG1314	pKC1139 derivative for gene replacement of *ytkR7*	This study
pTG1315	pSET152 derivative containing *ytkR7* for gene complementation	This study
pTG1316	pSET152 derivative containing *c10R6* for gene complementation	This study
pTG1317	pET-37b(+) derivative containing *ytkR7* for protein expression	This study
pTG1318	pET-37b(+) derivative containing *c10R6* for protein expression	This study
pTG1319	pET-28a(+) derivative containing *SHJG_8481* for protein expression	This study
pTG1320	pET-37b(+) derivative containing *Chte2144* for protein expression	This study
pTG1321	pET-37b(+) derivative containing *ETI84332.1* for protein expression	This study
pTG1322	pET-37b(+) derivative containing *lin2189* for protein expression	This study
pTG1323	pET-37b(+) derivative containing *SSDG_03674* for protein expression	This study
pTG1324	pET-37b(+) derivative containing *MA1133* for protein expression	This study
pTG1325	pET-37b(+) derivative containing *bmrR* for protein expression	This study
pTG1326	pET-37b(+) derivative containing *sbmC*for protein expression	This study
pTG1327	pET-37b(+) derivative containing *rob* for protein expression	This study

Supplementary Table 3 | X-ray data collection and refinement statistics

	lin2189 E157A E185L (5X5R)	lin2189 E157A E185L-YTM (5X5M)
**Data collection**		
Space group	*C*2	*C*2
Cell dimensions		
a, *b*, *c* (Å)	120.0, 48.7, 80.3	119.6, 49.3, 80.4
α, β, γ (°)	90, 125.5, 90	90.0, 125.0, 90.0
Resolution (Å)	50.00-1.65 (1.68-1.65)	50.00-1.21 (1.23-1.21)
R_{merge}	6.5 (72.8)	6.1 (36.9)
$I / \sigma I$	25.5 (1.78)	30.5 (4.8)
Completeness (%)	98.3 (98.8)	96.0 (84.2)
Redundancy	6.7 (6.7)	6.9 (6.6)
**Refinement**		
Resolution (Å)	26.41-1.65	26.48-1.21
No. unique reflections	45205	112031
R_{work} / R_{free}	0.20/0.24	0.18/0.19
No. of non-hydrogen atoms	3365	3921
Protein	3177	3386
Ligand/ion	N.A.	106
B-factors	29.22	19.93
Protein	28.96	19.04
Ligand/ion	N.A.	22.13
Solvent	33.54	26.41
R.m.s. deviations		
Bond lengths (Å)	0.008	0.006
Bond angles (°)	1.02	0.93

*Values in parentheses are for highest-resolution shell.

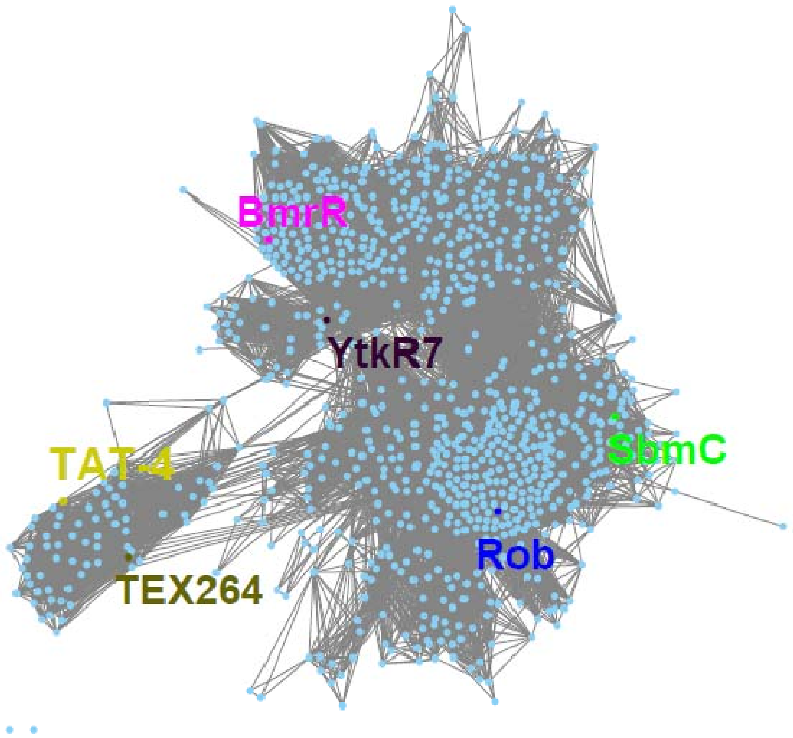

Supplementary Figure 1 | Sequence similarity network (SSN) analysis of GyrI-like small molecule binding domain containing proteins.

All the 12, 304 GyrI-like sequences and TEX264 homologues were first clustered, and the representatives of the clusters were then used to generate an SSN. The proteins mentioned include the *E. coli* SbmC (also designated as the DNA gyrase inhibitor GyrI, the prototype of GyrI-like proteins, P33012.1), the *E. coli* transcription factor Rob (NP_418813.1), the *Bacillus subtilis* multidrug transporter Bmr gene regulator BmrR (P39075.3), the *Streptomyces* sp. TP-A0356 YtkR7 (ADZ13561.1), the *Caenorhabditis elegans* TAT-4 (NP_495245.1), and the *Homo sapiens* testis expressed 264 (TEX264, NP_001123356.1).

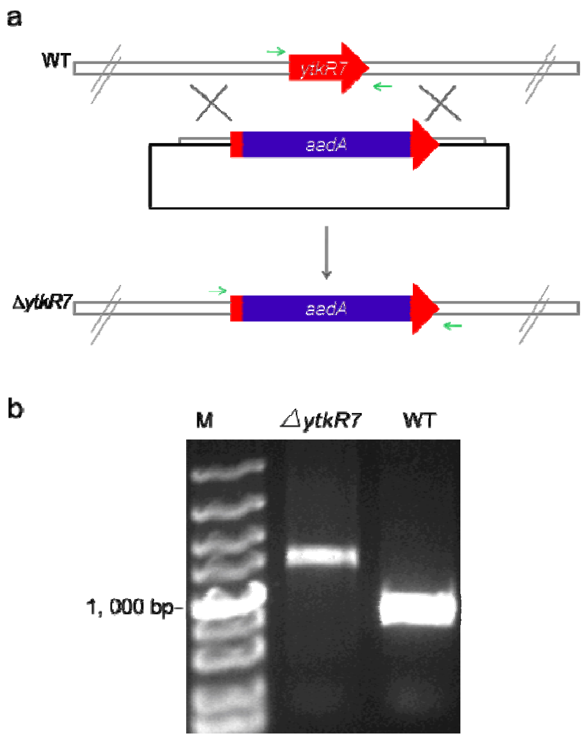

Supplementary Figure 2 | Construction and PCR verification of the *ytkR7* mutant.

(a) Illustration of the wild type *Streptomyces* sp. TP-A0356 *ytkR7* gene is replaced with the *aadA* disruption cassette. (b) PCR verification of the *ytkR7* mutant. The wild type *ytkR7* PCR products, 746 bp; and the *ytkR7* mutant PCR products, 1 610 bp.

a

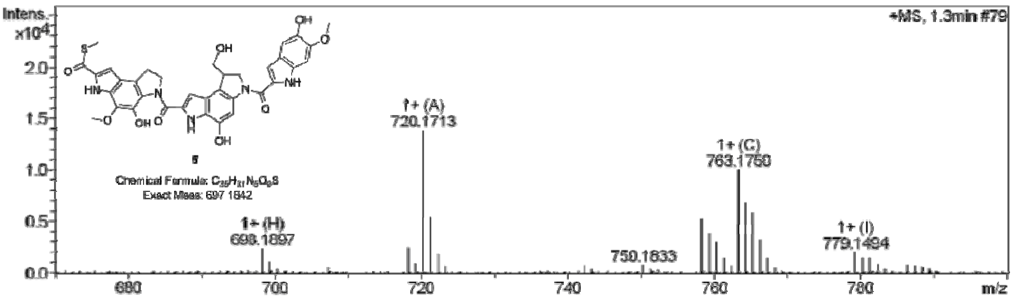

Meas. m/z	#	Formula	Score	m/z	err [ppm]	Mean err [ppm]	mSigma	rdb	e⁻ Conf	N-Rule
698.1897	1	C 35 H 32 N 5 O 9 S	60.35	698.1915	2.6	3.2	27.6	22.5	even	ok
720.1713	1	C 35 H 31 N 5 Na O 9 S	100.00	720.1735	3.0	3.3	12.0	22.5	even	ok

b

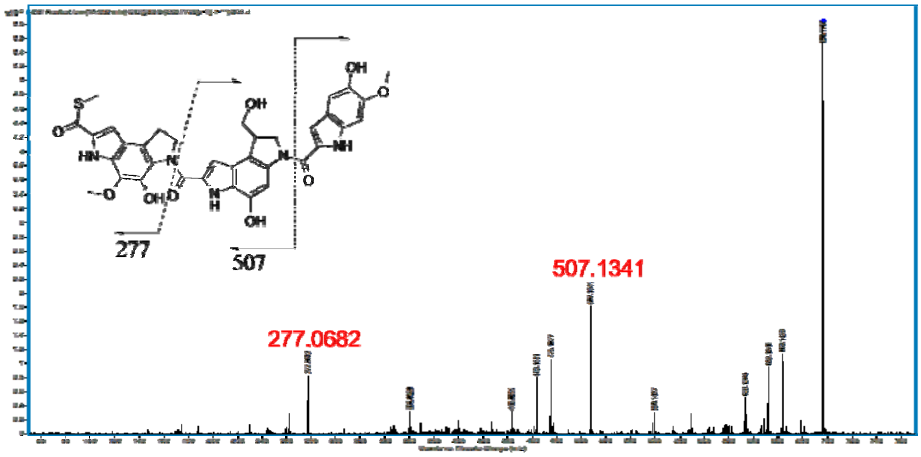

Supplementary Figure 3 | MS analyses of compound 5.

(a) HR-MS analysis. (b) MS/MS analysis.

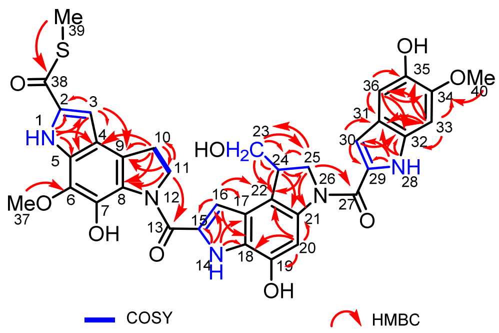

Position	δC, type	δH (mult., *J* in Hz)	H-H COSY	HMBC（H→C)
1-NH		13.75, (s, 1H)	H-3	C-2,3,4,5
2	133.4, C			
3	108.4, CH	7.56, (d, 1H, 1.5)	1-NH	C-2,4,5,9
4	118.7, C			
5	136.0, C			
6	134.6, C			
7	141.1, C			
8	129.1, C			
9	122.1, C			
10	28.4, CH_2	3.25, (m, 2H)	H-11	C-4,8,9,11
11	54.1, CH_2	4.55, (m, 1H)	H-10	C-8,9,10,13
		4.66, (m, 1H)		
13	162.1, C			
14-NH		13.45, (s, 1H)	H-16	C-15,16,17,18
15	131.4, C			
16	106.5, CH	7.40, (s, 1H)	14-NH	C-15,17,18,22
17	125.8, C			
18	127.1, C			
19	139.8, C			
20	102.2, C	6.93, (s, 1H)※		C-18,19,21,22※
21	125.9, C			
22	115.1, C			
23	64.9, CH_2	4.19, (m, 1H)	H-24,25	C-22,24,25
		4.50, (m, 1H)		
24	44.9, CH	4.26, (m, 1H)	H-23,25	C-21,22,23,25
25	55.5, CH_2	4.88, (m, 1H)	H-23,24	C-21,22,23,24,27
		5.02, (m, 1H)		
27	160.8, C			
28-NH		12.45, (s, 1H)	H-30	C-29,30,31,32
29	131.3, C			
30	105.9, CH	7.19, (s, 1H)	28-NH	C-29,31,32
31	122.8, C			
32	132.1, C			

33	106.7, C	7.65, (s, 1H)	C-31,32,34,35,36
34	149.5, C		
35	144.4, C		
36	94.7, C	7.29, (s, 1H)	C-31,32,33,34,35
37	60.5, CH_3	4.06, (s, 3H)	C-6
38	183.6, C		
39	11.2, CH_3	2.52, (m, 3H)	C-38
40	56.1, CH_3	3.86, (s, 3H)	C-34

In pyridine-d_5, 500 MHz for ^{1}H and 125 MHz for ^{13}C NMR

※: not observed in pyridine-d_5, but observed in DMSO- d_6

(a) ^{1}H NMR spectrum

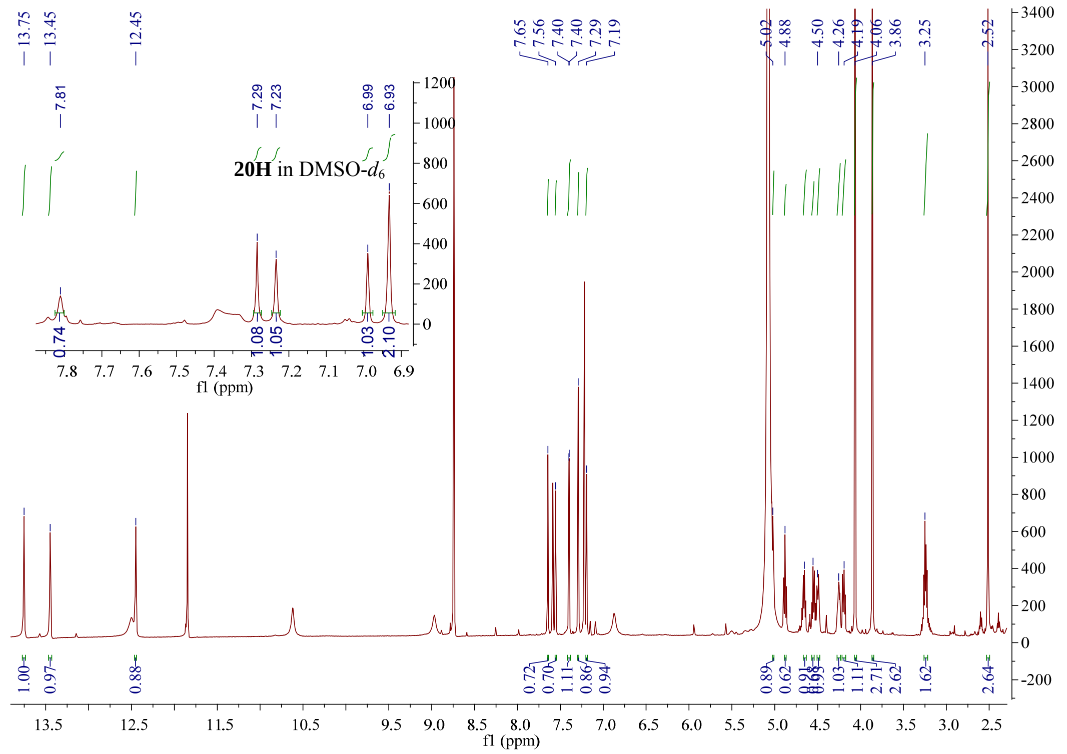

(b) ^{13}C NMR spectrum

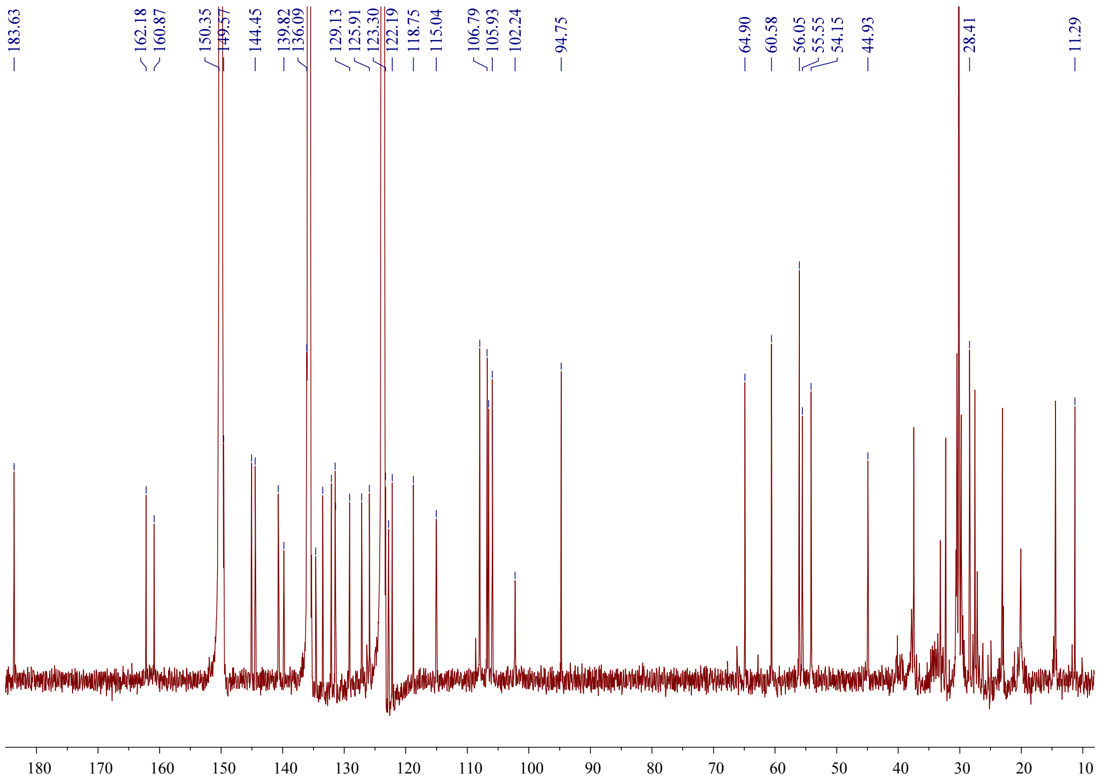

(c) DEPT spectrum

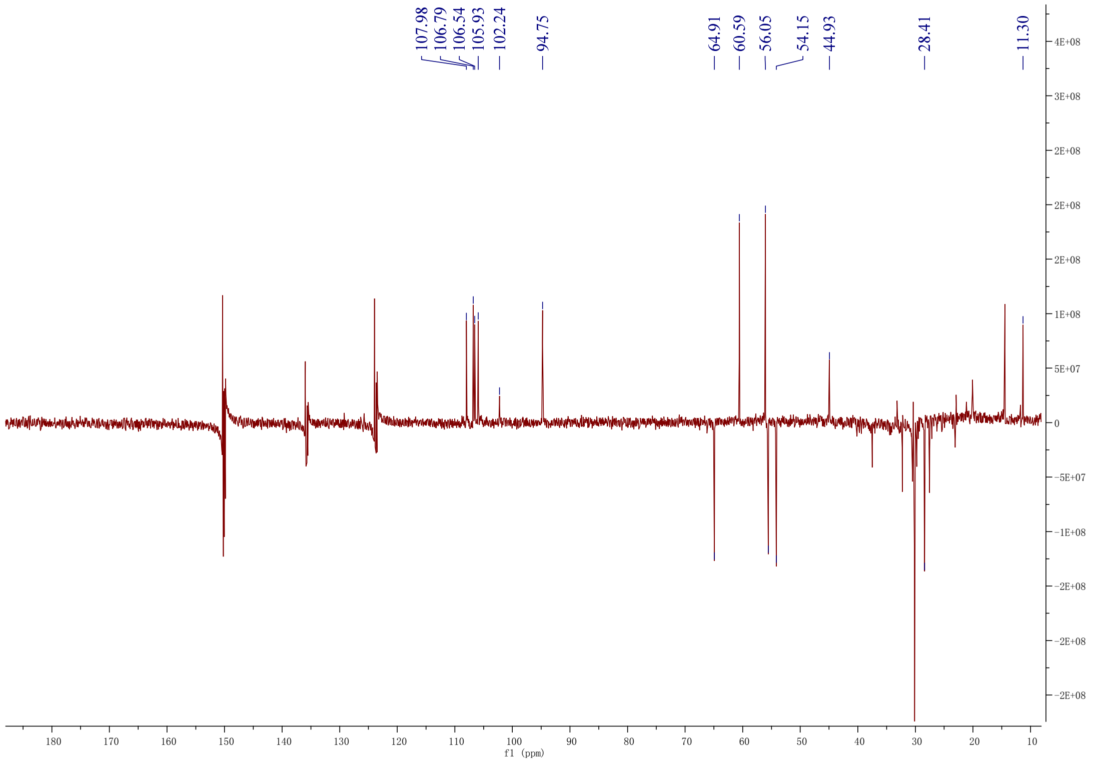

(d) ^{1}H-^{1}H COSY spectrum

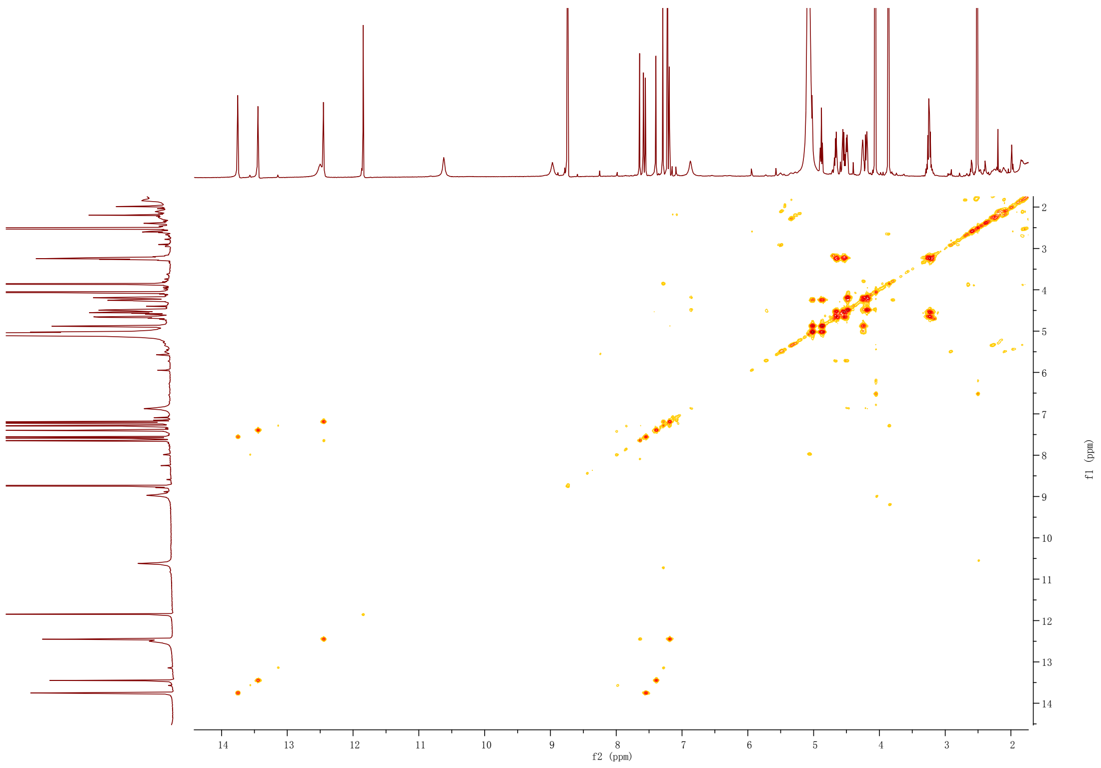

(e) HSQC spectrum

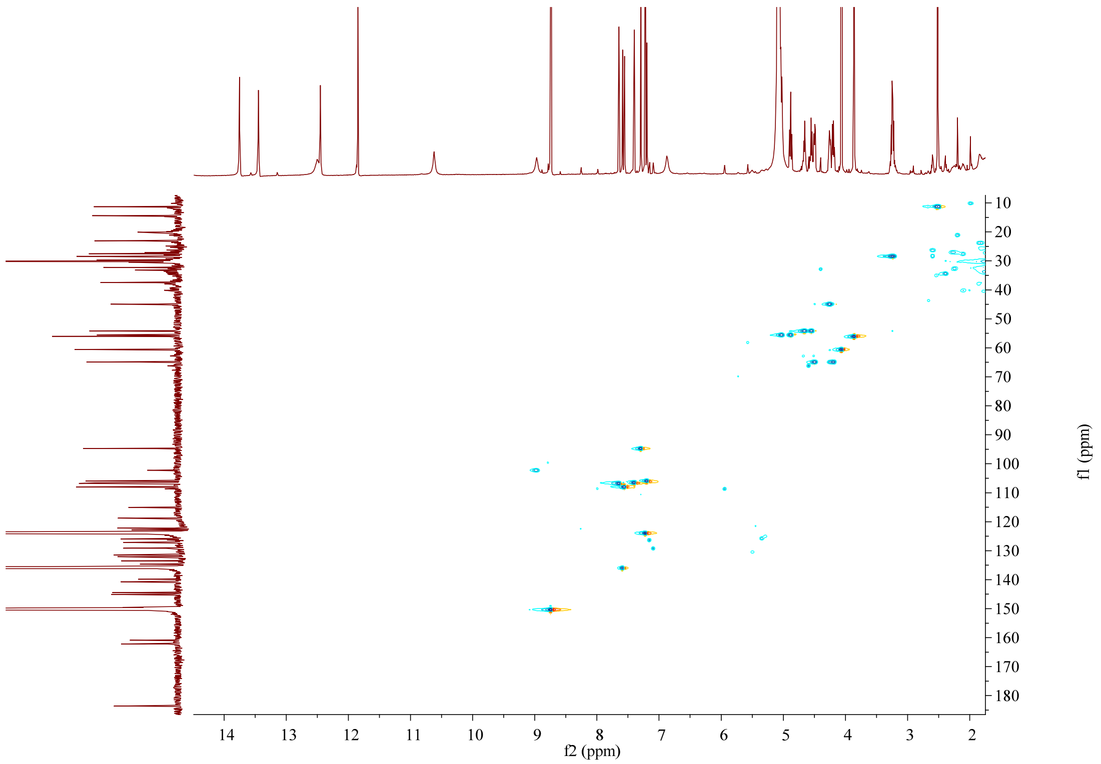

(f) HMBC spectrum

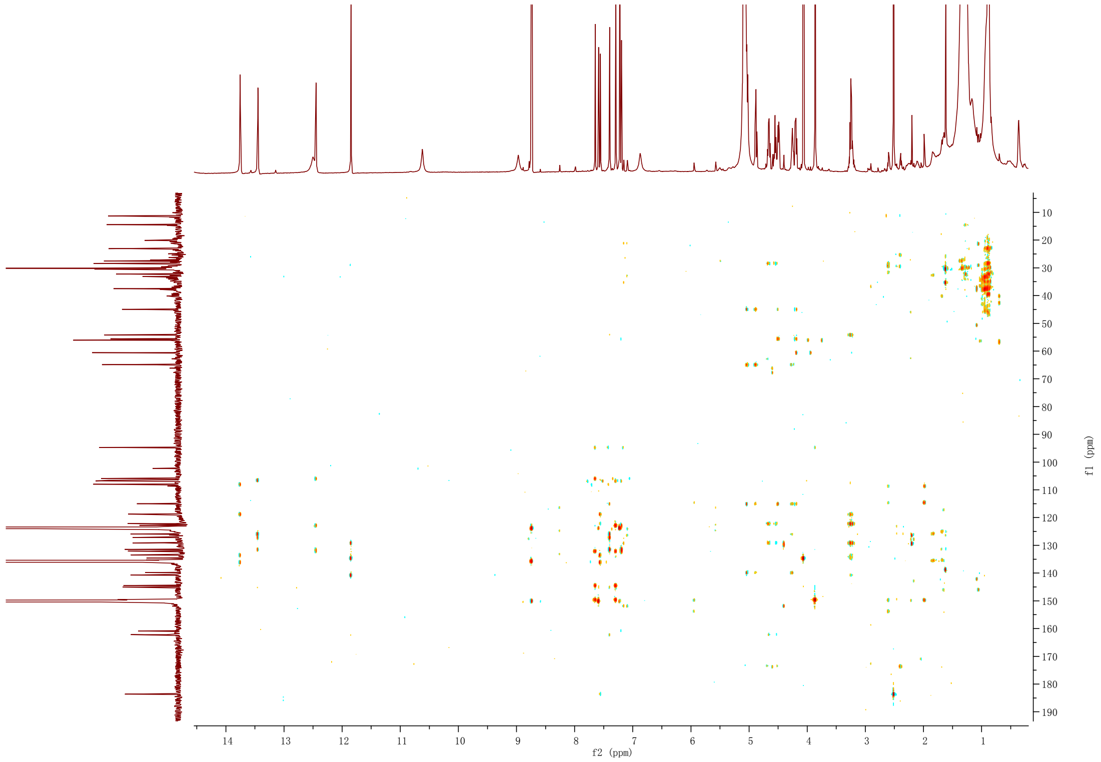

Supplementary Figure 4 | NMR spectroscopic data of compound 5.

(a) ^{1}H NMR spectrum. (b) ^{13}C NMR spectrum. (c) DEPT spectrum. (d) ^{1}H-^{1}H COSY spectrum. (e) HSQC spectrum. (f) HMBC spectrum.

a

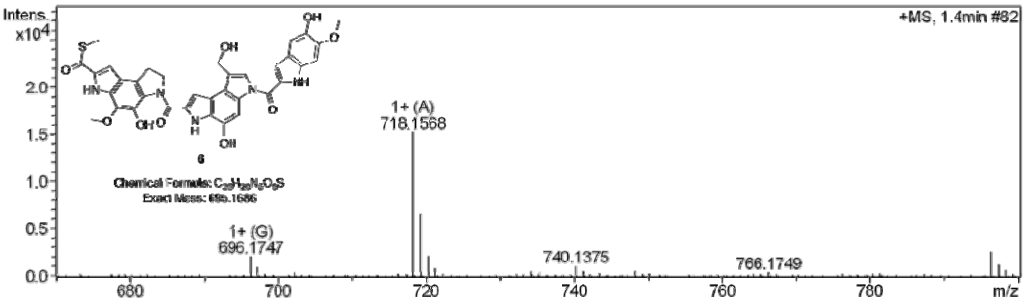

Meas. m/z	#	Formula	Score	m/z	err [ppm]	Mean err [ppm]	mSigma	rdb	e⁻ Conf	N-Rule
696.1747	1	C 35 H 30 N 5 O 9 S	100.00	696.1759	1.7	1.9	24.1	23.5	even	ok
718.1568	1	C 35 H 29 N 5 Na O 9 S	100.00	718.1578	1.4	2.0	15.1	23.5	even	ok

b

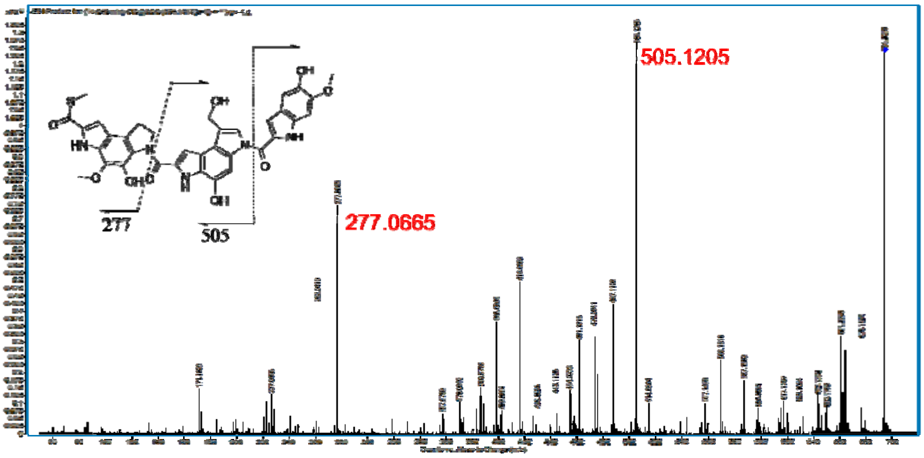

Supplementary Figure 5 | MS analyses of compound 6.

(a) HR-MS analysis. (b) MS/MS analysis.

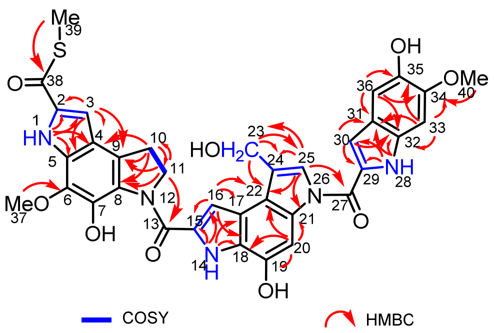

Position	δC, type	δH (mult., *J* in Hz)	H-H COSY	HMBC（H→C)
1-NH		13.65, (s, 1H)	H-3	C-2,3,4,5
2	133.6, C			
3	108.3, CH	7.54, (s, 1H)	1-NH	C-2,4,5,9
4	118.8, C			
5	135.6, C			
6	134.6, C			
7	140.7, C			
8	129.6, C			
9	121.8, C			
10	28.8, CH_2	3.22, (t, 2H, 8.0)	H-11	C-4,8,9,11
11	54.6, CH_2	4.76, (t, 2H, 8.0)	H-10	C-8,9,10,13
13	162.3, C			
14-NH		13.32, (s, 1H)	H-16	C-15,16,17,18
15	130.3, C			
16	109.1, CH	8.00, (br.s, 1H)	14-NH	C-15,17,18,22
17	122.0, C			
18	127.7, C			
19	144.2, C			
20	100.1, CH	8.78, (s, 1H)		C-18,19,21,22
21	133.1, C			
22	116.8, C			
23	58.5, CH_2	5.57, (s, 2H)	H-25	C-22,24,25
24	123.4, C			
25	122.7, CH	8.24, (s, 1H)	H-23	C-21,22,23,24,27
27	162.6, C			
28-NH		13.01, (s, 1H)	H-30	C-29,30,31,32
29	129.6, C			
30	111.0, CH	7.29, (s, 1H)	28-NH	C-29,31,32
31	122.4, C			
32	133.9, C			
33	107.1, CH	7.58, (s, 1H)		C-31,32,34,35
34	150.5, C			
35	145.1, C			
36	95.1, CH	7.29, (s, 1H)		C-31,32,34,35

37	61.0, CH_3	4.09, (s, 3H)	C-6
38	184.0, C		
39	11.6, CH_3	2.52, (s, 3H)	C-38
40	56.4, CH_3	3.88, (s, 3H)	C-34

In pyridine-d_5, 500 MHz for ^{1}H and 125 MHz for ^{13}C NMR

(a) ^{1}H NMR spectrum

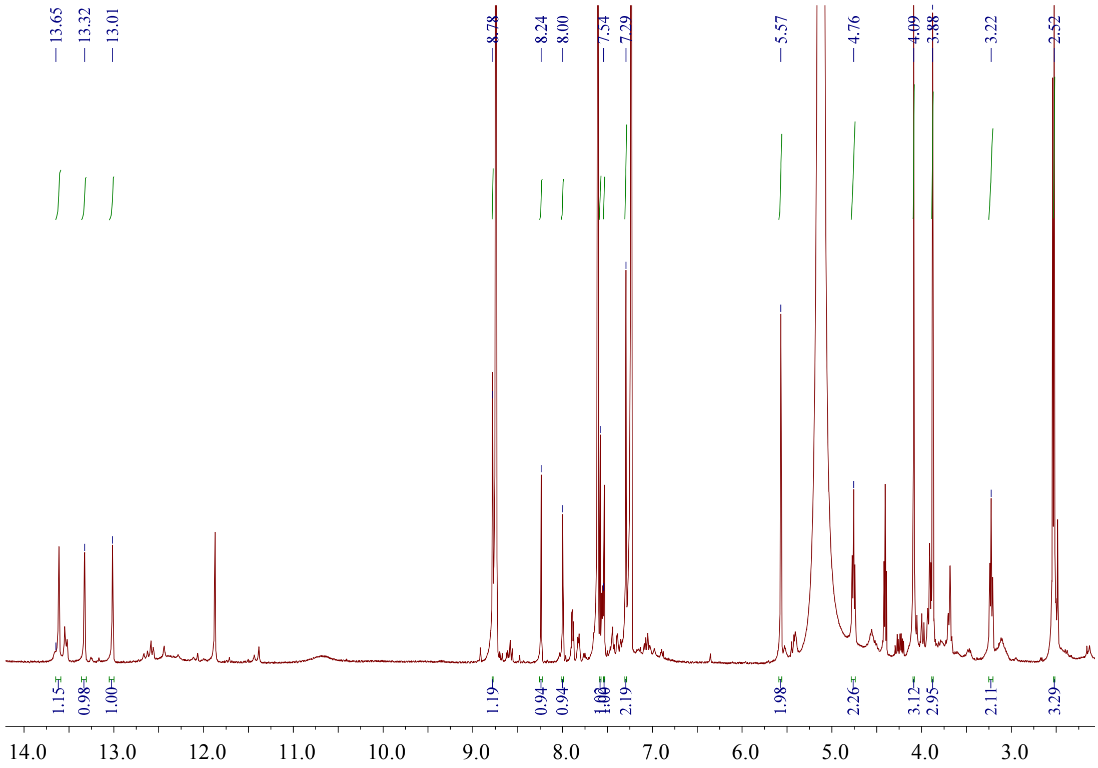

(b) ^{13}C NMR spectrum

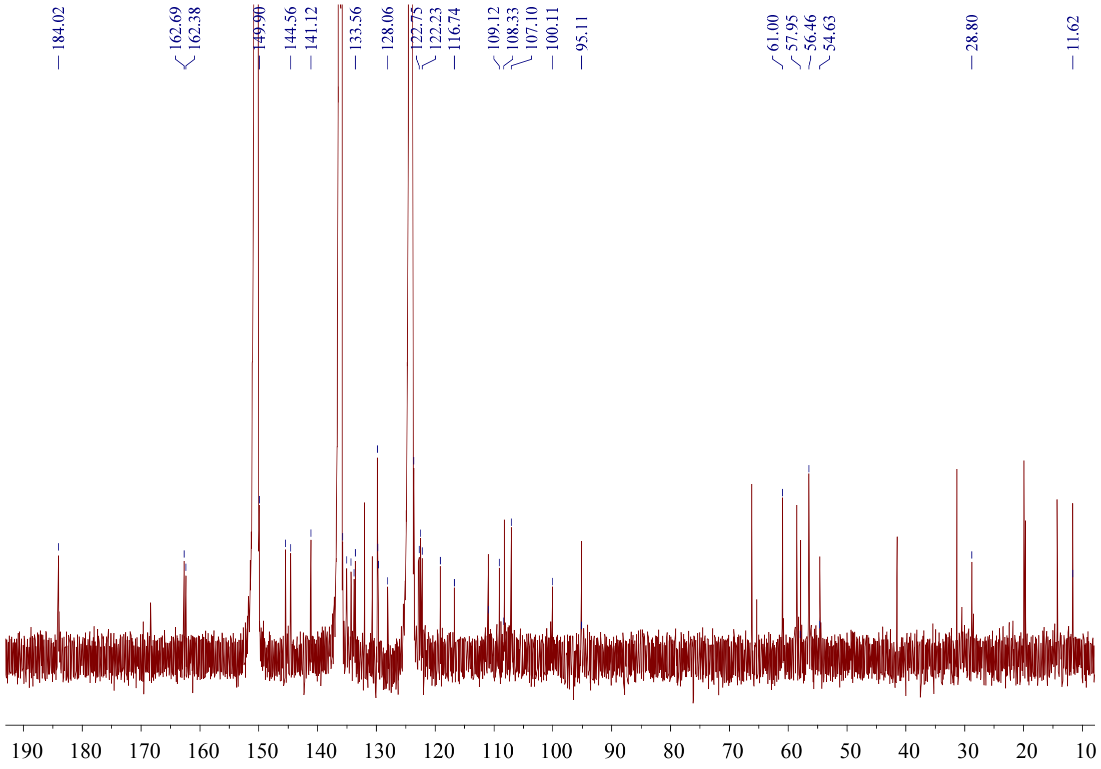

(c) DEPT spectrum

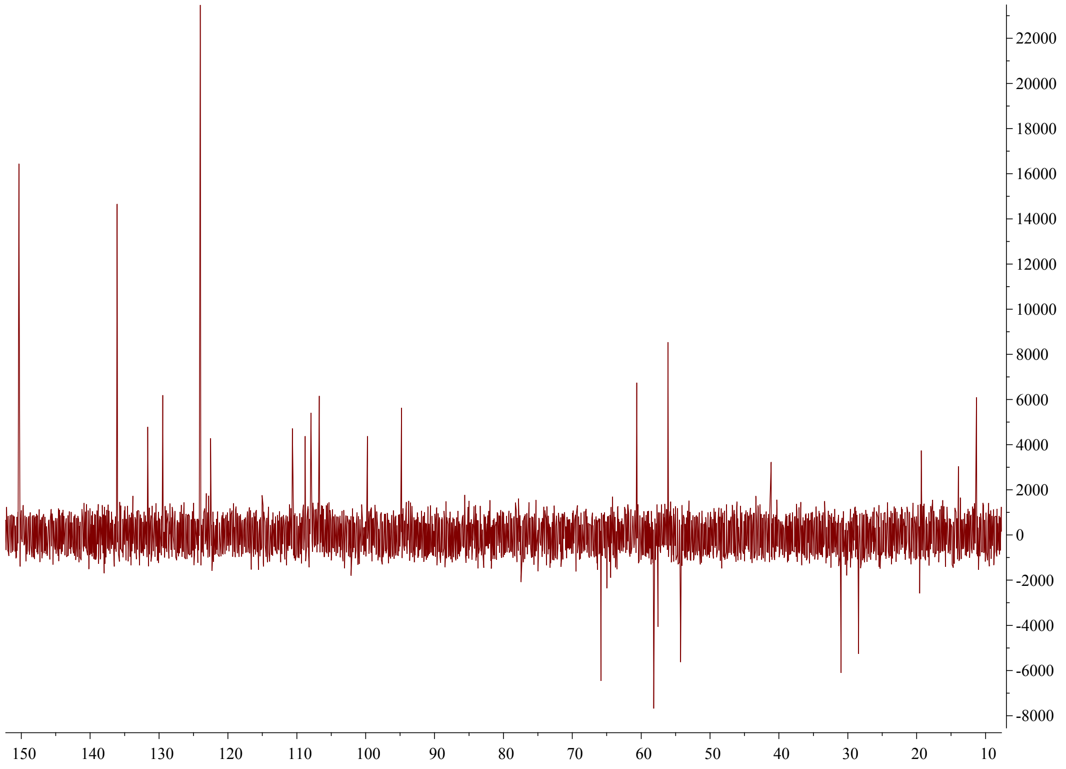

(d) ^{1}H-^{1}H COSY spectrum

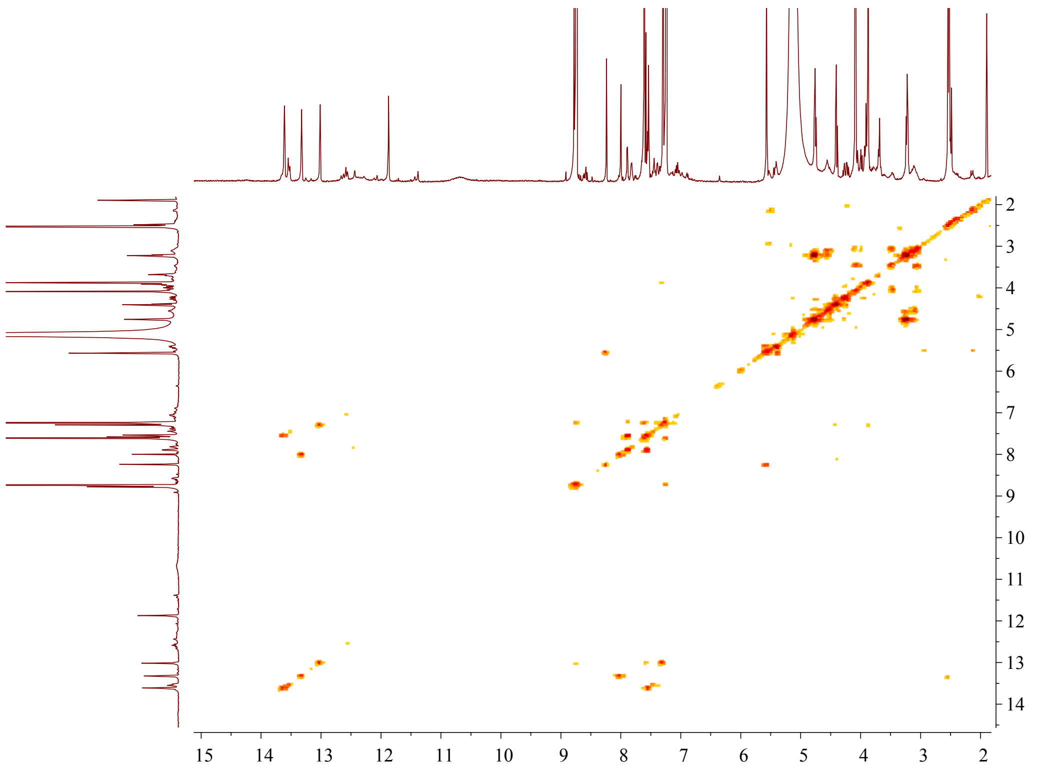

(e) HSQC spectrum

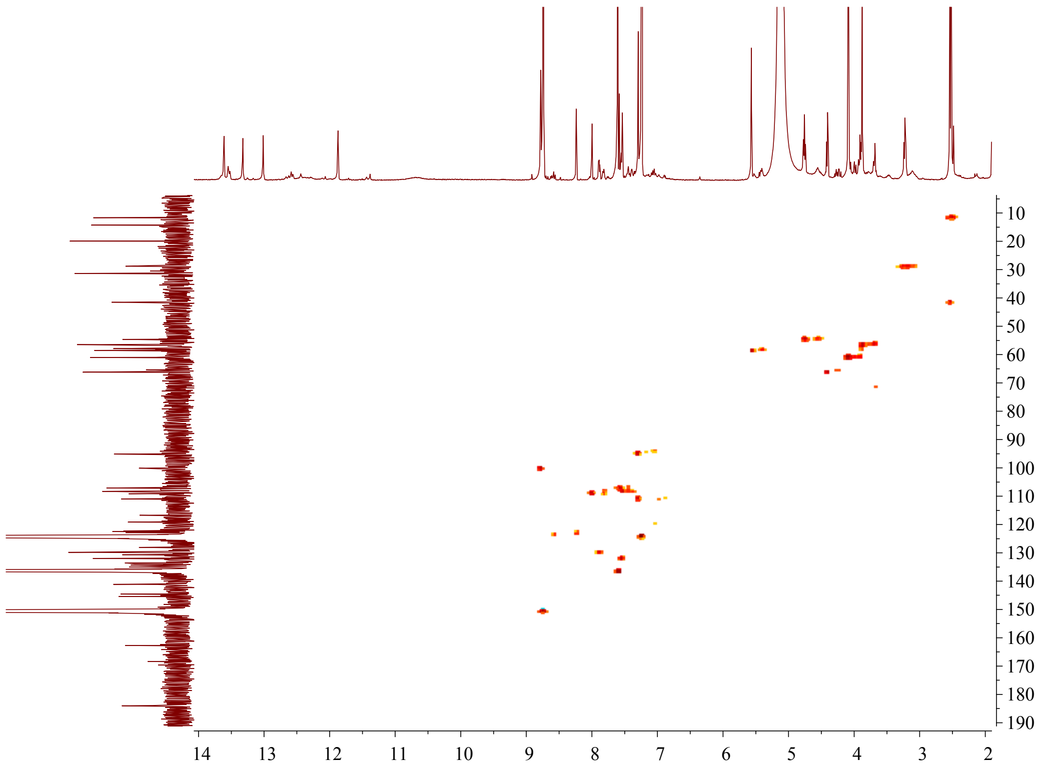

(f) HMBC spectrum

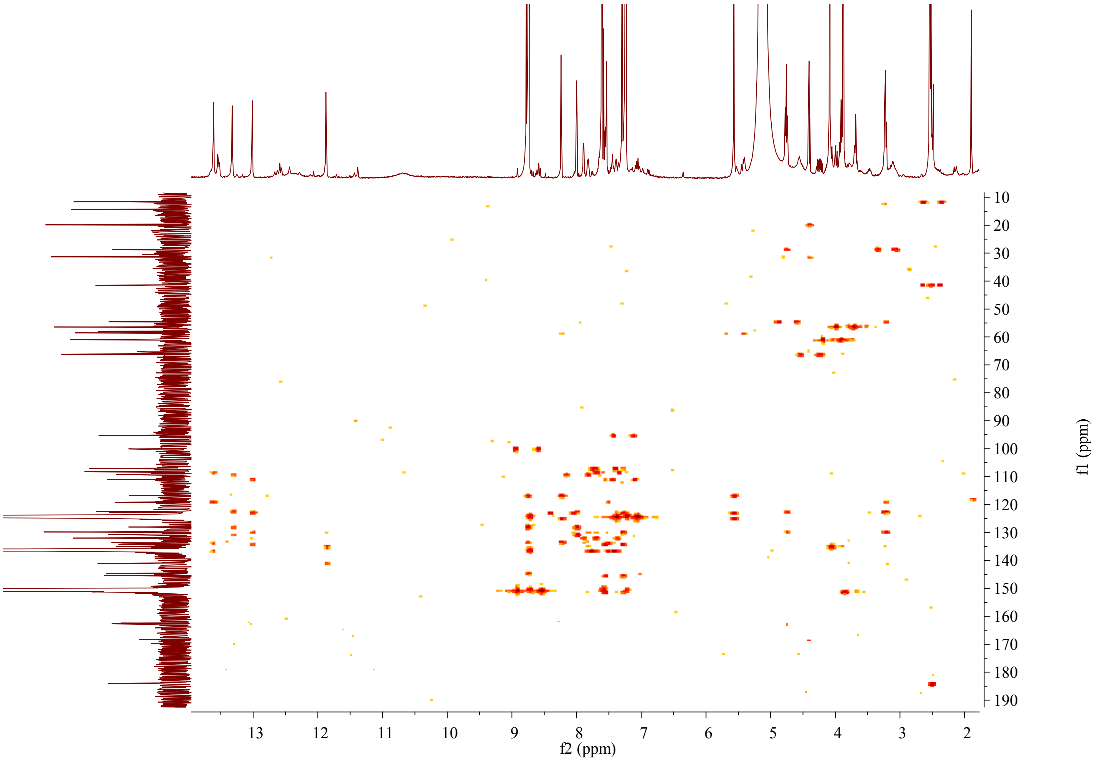

Supplementary Figure 6 | NMR spectroscopic data of compound 6.

(a) ^1H NMR spectrum. (b) ^{13}C NMR spectrum. (c) DEPT spectrum. (d) ^1H-^1H COSY spectrum. (e) HSQC spectrum. (f) HMBC spectrum.

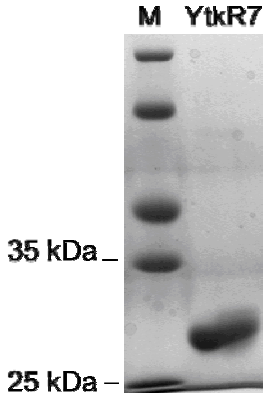

Supplementary Figure 7 | SDS-PAGE analysis of the purified YtkR7 protein

a

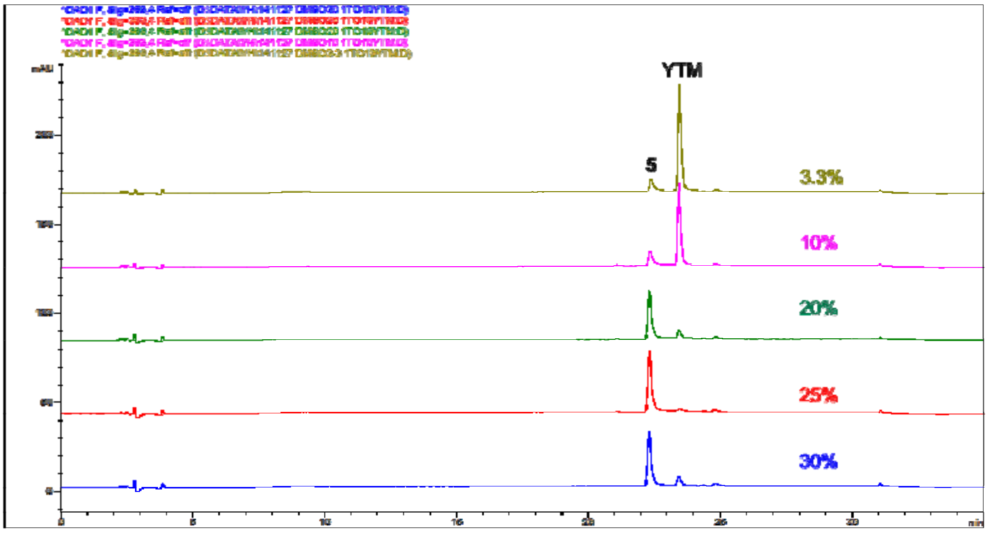

b

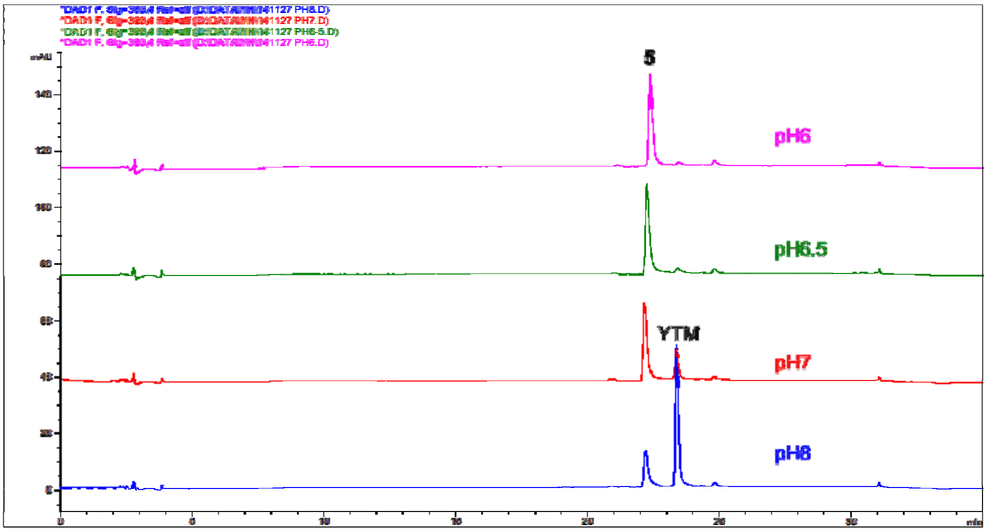

Supplementary Figure 8 | Effects of DMSO and pH conditions on YTM hydrolysis catalyzed by YtkR7.

(a) The effects of the concentration of DMSO on the YTM hydrolysis. (b) The effects of pH on the YTM hydrolysis. HPLC analysis with UV detection at 383 nm.

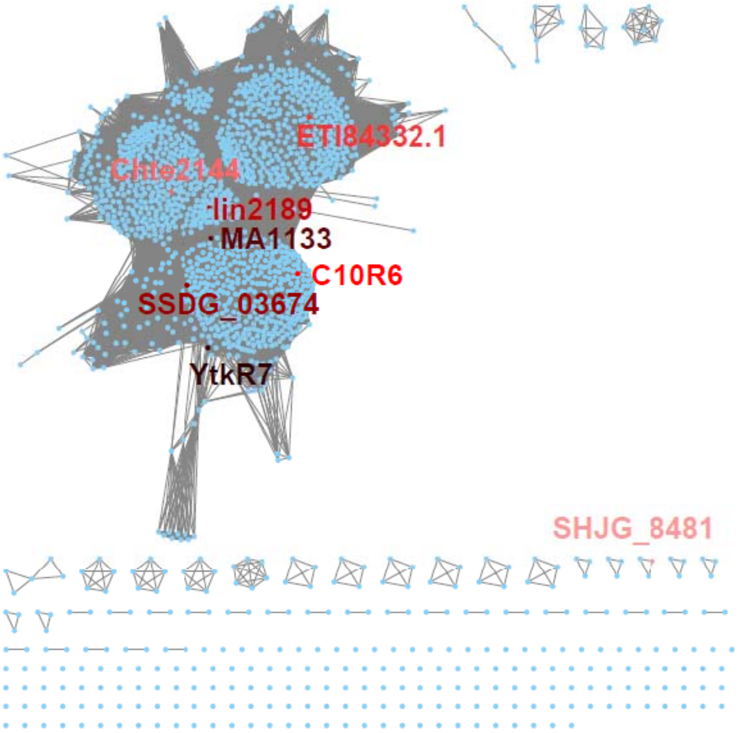

Supplementary Figure 9 | SSN analysis of CCHs.

The eight CCHs characterized in the text are YtkR7, SHJG_8481 from *S. hygroscopicus* subsp. jinggangensis 5008 (Identity, 36%; E value, 3e-29), Chte2144 from *Clostridium thermocellum* ATCC 27405 (Identity, 31%; E value, 3e-32), ETI84332.1 from *Streptococcus anginosus* DORA_7 (of the human microbiota; Identity, 33%; E value, 4e-39), C10R6 from the CC-1065 producer *S. zelensis* NRRL 11183 (GenBank KY379149; Identity, 36%; E value, 3e-42), lin2189 from *Listeria innocua* Clip11262 (Identity, 36%; E value, 3e-44), SSDG_03674 from *S. pristinaespiralis* ATCC 25486 (Identity, 40%; E value, 3e-47), and MA1133 from *Methanosarcina acetivorans* C2A (Identity, 42%; E value, 8e-58). The label color from light to dark corresponds to the identity (with YtkR7) from low to high.

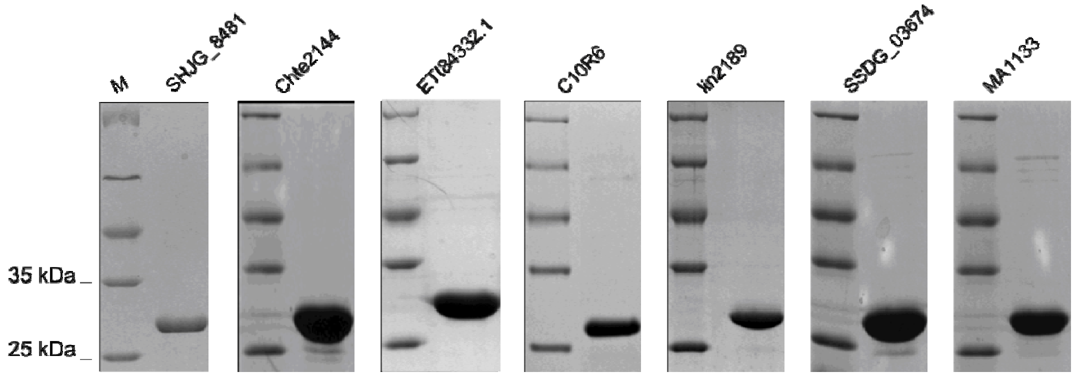

Supplementary Figure 10 | SDS-PAGE analyses of purified SHJG_8481, Chte2144, ETI84332.1, C10R6, lin2189, SSDG_03674 and MA1133 proteins

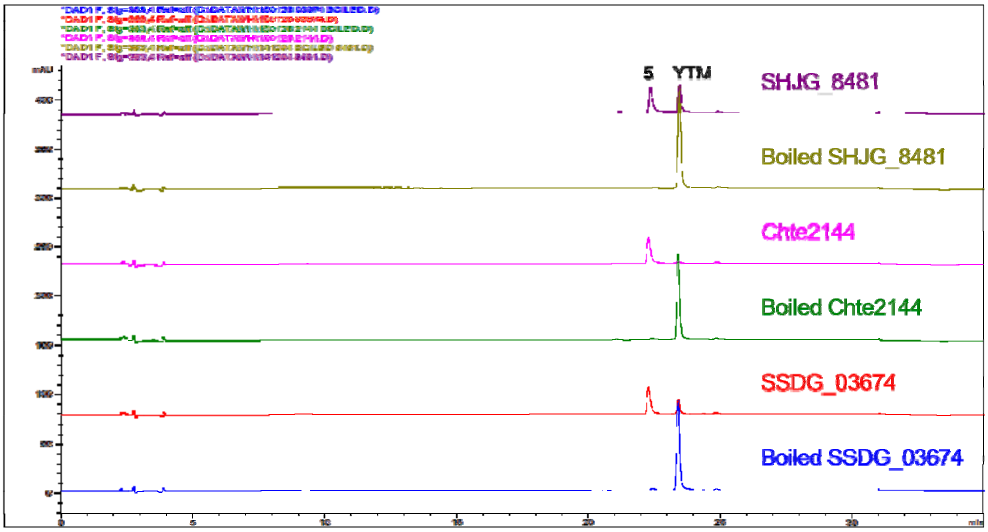

Supplementary Figure 11 | YTM hydrolysis catalyzed by SHJG_8481, Chte2144 and SSDG_03674.

HPLC analysis with UV detection at 383 nm.

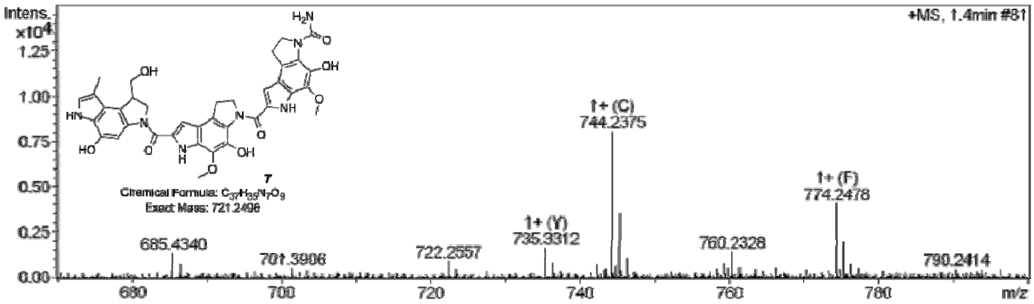

Meas. m/z	#	Formula	Score	m/z	err [ppm]	Mean err [ppm]	mSigma	rdb	e⁻ Conf	N-Rule
722.2557	1	C 37 H 36 N 7 O 9	100.00	722.2569	1.6	1.9	46.5	23.5	even	ok
744.2375	1	C 37 H 35 N 7 Na O 9	100.00	744.2388	1.8	2.4	16.0	23.5	even	ok

Supplementary Figure 12 | HR-MS analysis of compound 7

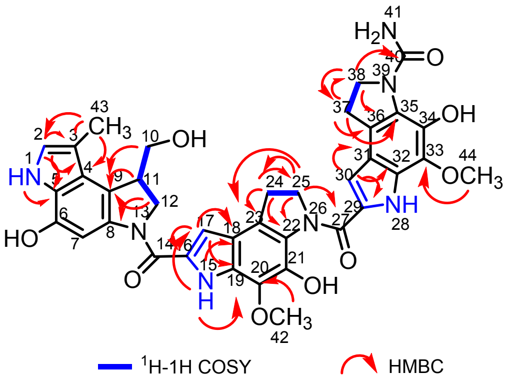

Position	δC, type	δH (mult., *J* in Hz)	H-H COSY	HMBC（H→C)
1-NH		11.25 (s, 1H)	H-2	C-5
2	123.8, CH	6.99 (s, 1H)	1-NH, 43	C-3,4,5
3	108.8, C			
4	124.6, C			
5	132.4, C			
6	142.7, C			
7	97.5, CH	6.89 (m, 1H)		
8	135.9, C			
9	112.9, C			
10	64.5, CH_2	3.14 (m, 1H)	H-10′	C-9
		3.66 (m, 1H)	H-10	
11	42.7, CH	3.66 (m, 1H)	H-10,10′	C-9
12	54.5, CH_2	4.47 (d, 1H)	H-11	C-8,9
		4.52 (d, 1H)	H-11	
14	159.2, C			
15-NH		11.25 (s, 1H)	H-17	C-17,18,19
16	124.6, C			
17	103.7, CH	6.99 (s, 1H)	15-NH	C-16,18,19
18	117.9, C			
19	129.9, C			
20	133.1, C			
21	137.6 C			
21-OH		10.91 (s, 1H, OH)		
22	127.0, C			
23	120.8, C			
24	27.7, CH_2	3.37 (m, 2H)	H-25	C-22,23,25
25	53.4, CH_2	4.69 (t, 2H, 7.8)	H-24	C-22,23,24,27
27	160.6, C			
28-NH		10.91 (s, 1H)	H-30	
29	129.1, C			
30	106.2, CH	7.06 (s, 1H)	28-NH	C-29,31,32
31	117.2, C			

32	129.5, C			
33	132.4, C			
34	137.6, C			
34-OH		12.92 (s, 1H, OH)		
35	127.8, C			
36	118.2, C			
37	26.5, CH_2	3.24 (t, 2H, 8.4)	H-38	C-35,36,38
38	49.4, CH_2	4.03 (t, 2H, 8.4)	H-37	C-36,37,40
40	157.6, C			
41-NH_2		6.89 (s, 2H)		
42	60.3, CH_3	3.89 (s, 3H)		C-20
43	11.2, CH_3	2.35 (s, 3H)	H-2	C-2,3,4
44	60.0, CH_3	3.83 (s, 3H)		C-33

In DMSO-d_6, 500 MHz for ^1H and 125 MHz for ^{13}C NMR

(a) ^1H NMR spectrum

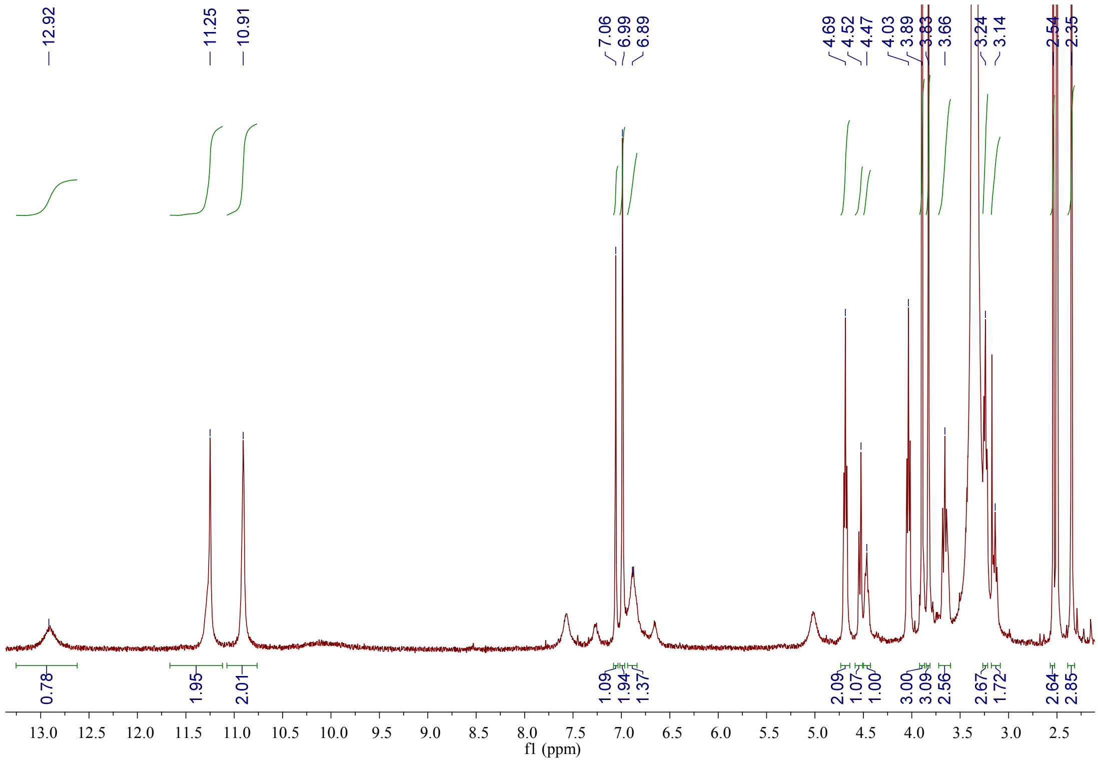

(b) ^{13}C NMR spectrum

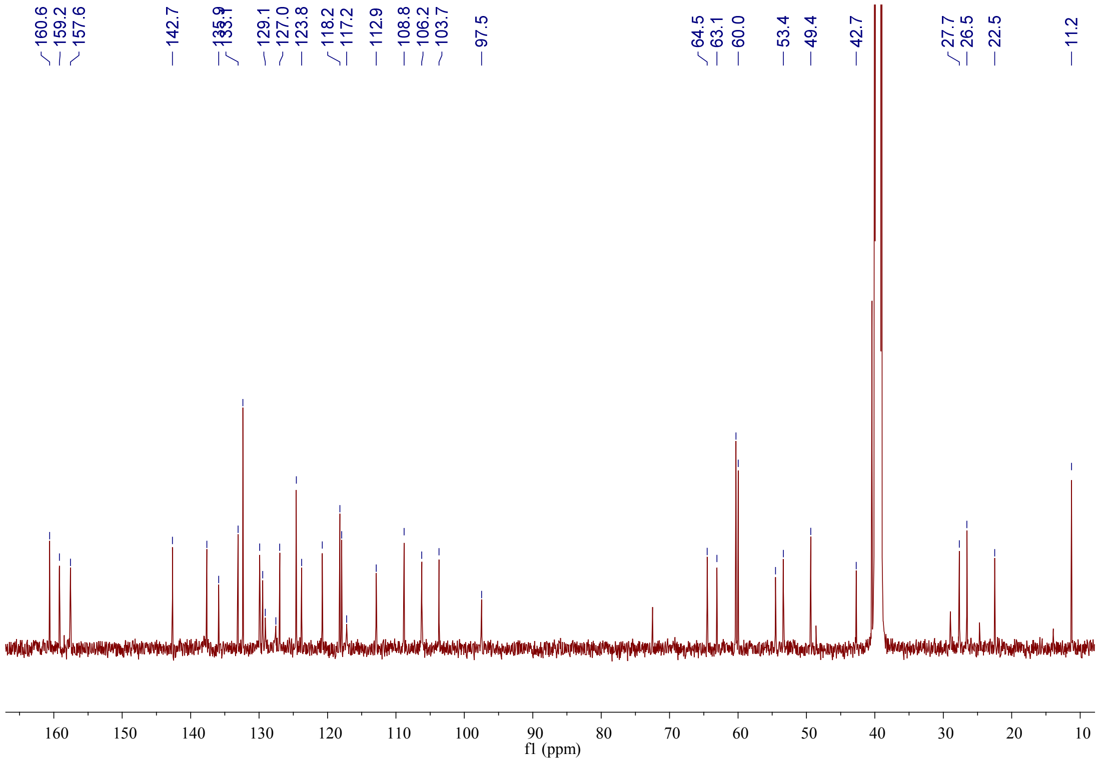

(c) DEPT spectrum

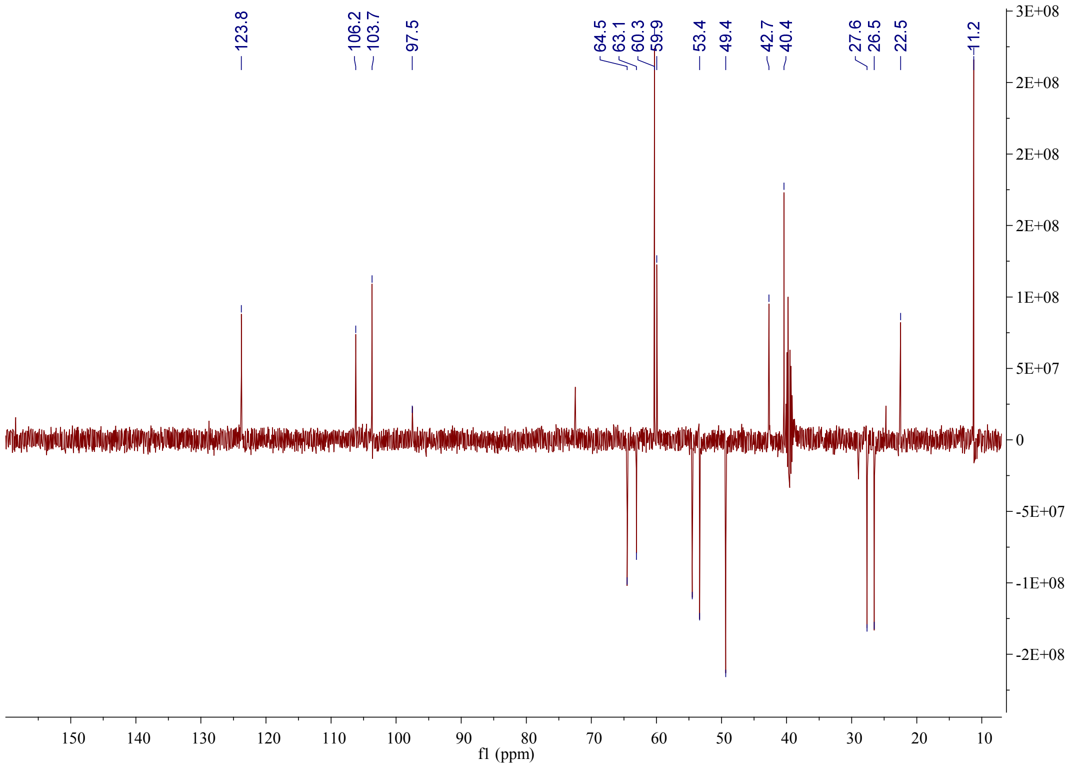

(d) ^{1}H-^{1}H COSY spectrum

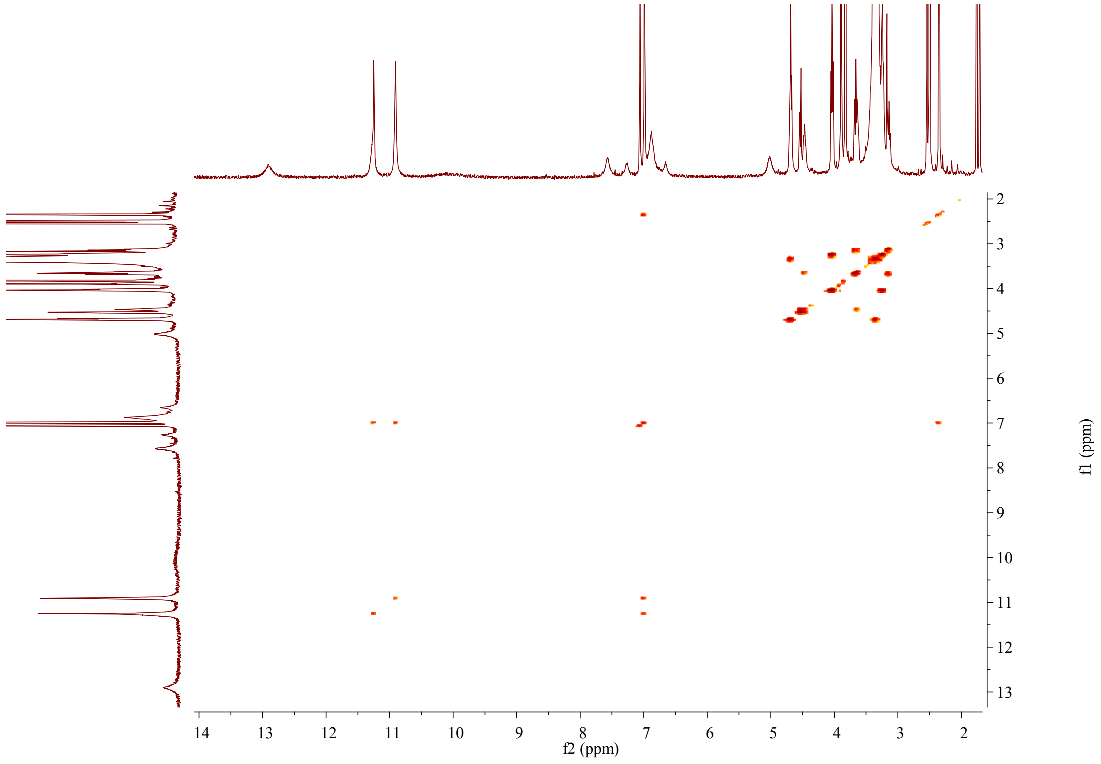

(e) HSQC spectrum

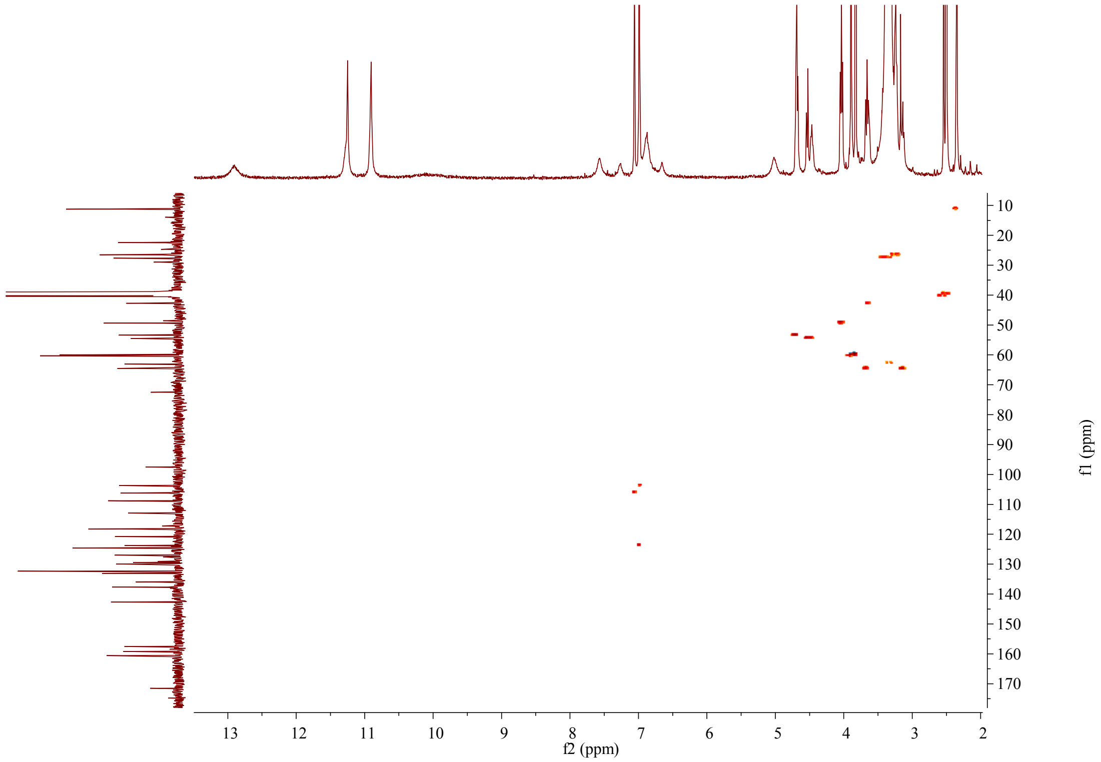

(f) HMBC spectrum

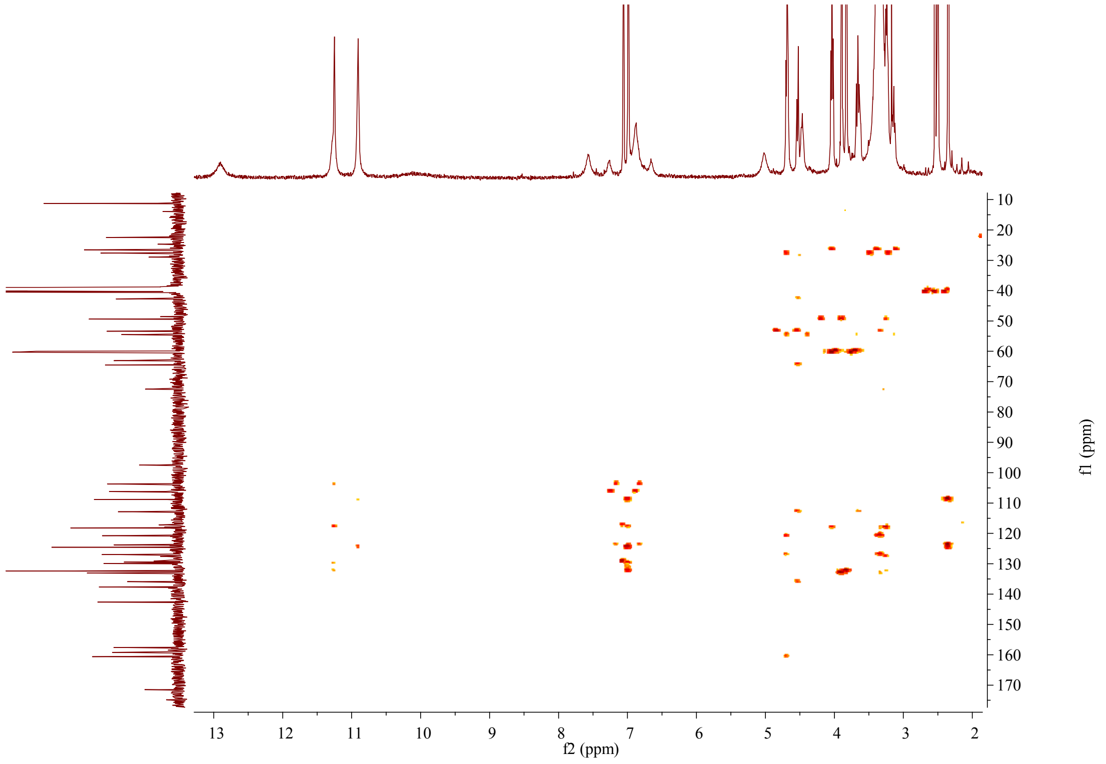

Supplementary Figure 13 | NMR spectroscopic data of compound 7.

(a) ^{1}H NMR spectrum. (b) ^{13}C NMR spectrum. (c) DEPT spectrum. (d) ^{1}H-^{1}H COSY spectrum. (e) HSQC spectrum. (f) HMBC spectrum.

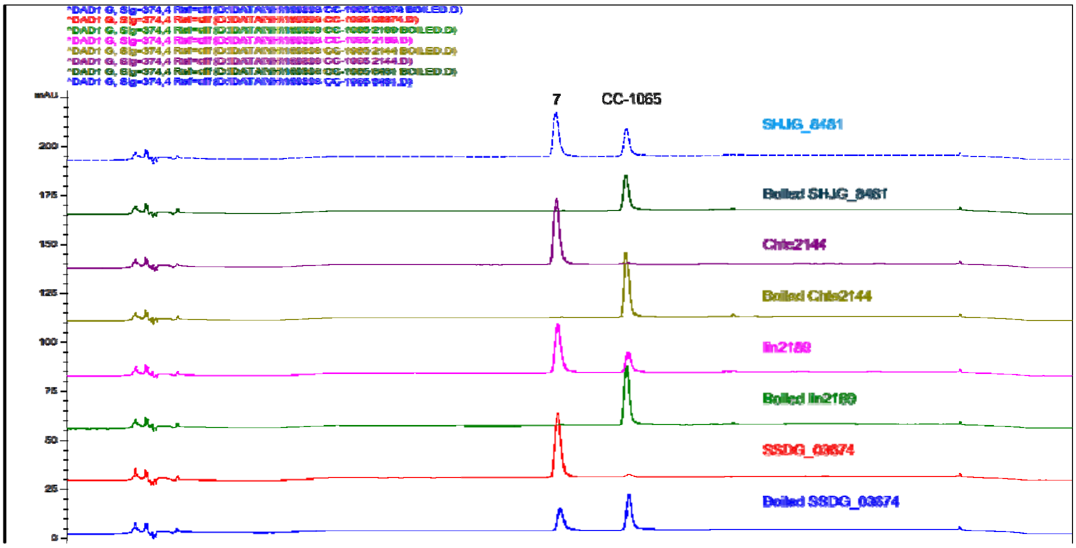

Supplementary Figure 14 | CC-1065 hydrolysis catalyzed by SSDG_03674, lin2189, Chte2144 and SHJG_8481.

HPLC analysis with UV detection at 374 nm.

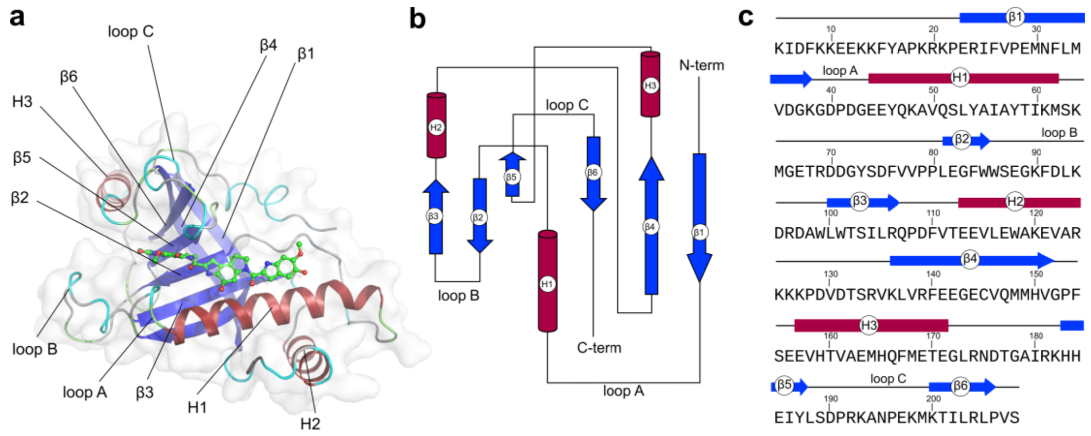

Supplementary Figure 15 | The topology of lin2189.

(a) The overall 3D structure of lin2189. Green ball-and-stick, the substrate molecule. Red cartoon, α-helices; blue cartoon, six β-strands. (b) The 2D diagram of lin2189. (c) The primary and secondary structure of lin2189.

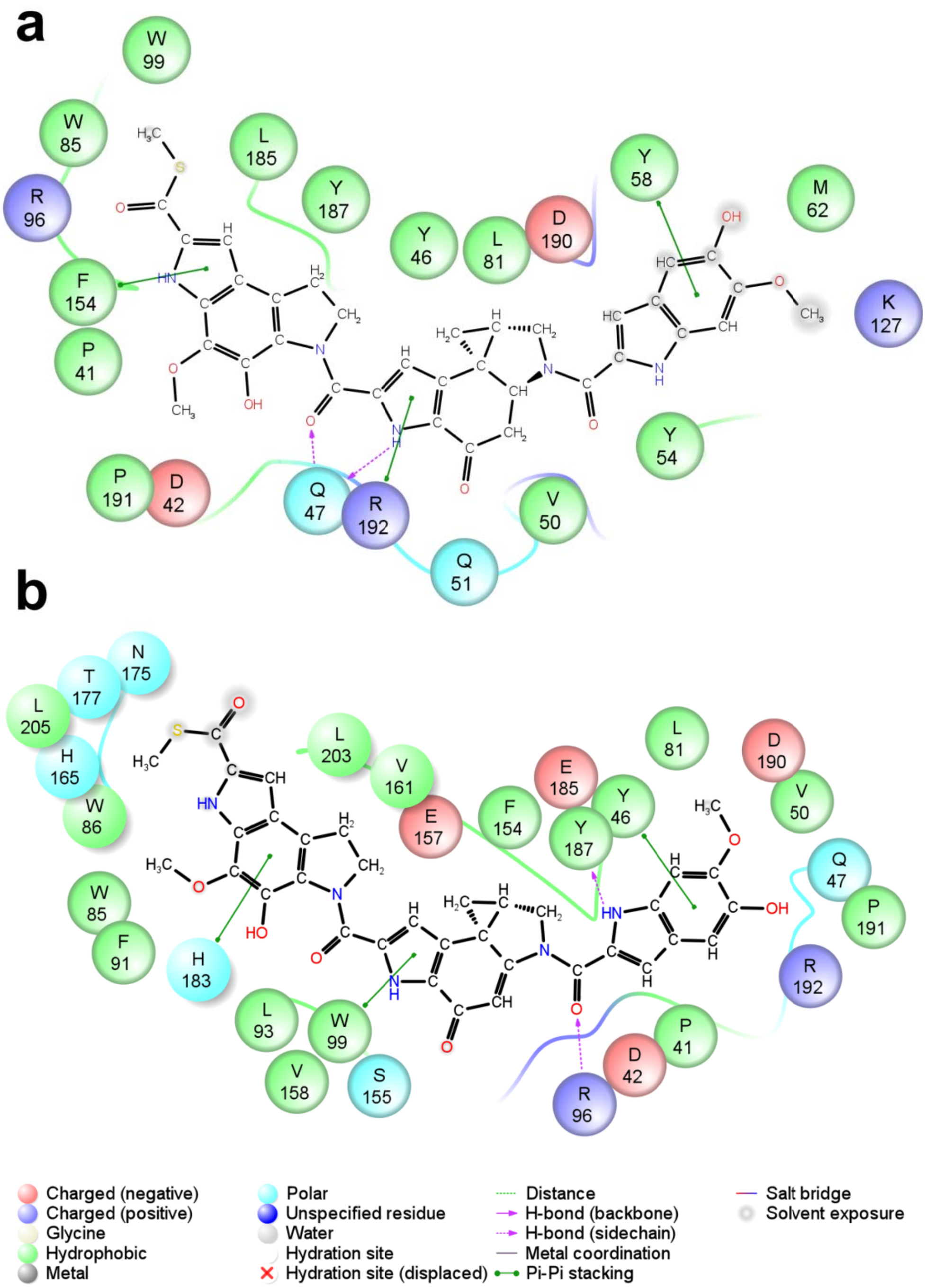

Supplementary Figure 16 | The 2D interaction diagrams for (a) substrate observed in the crystal structure and (b) substrate binding mode obtained from docking.

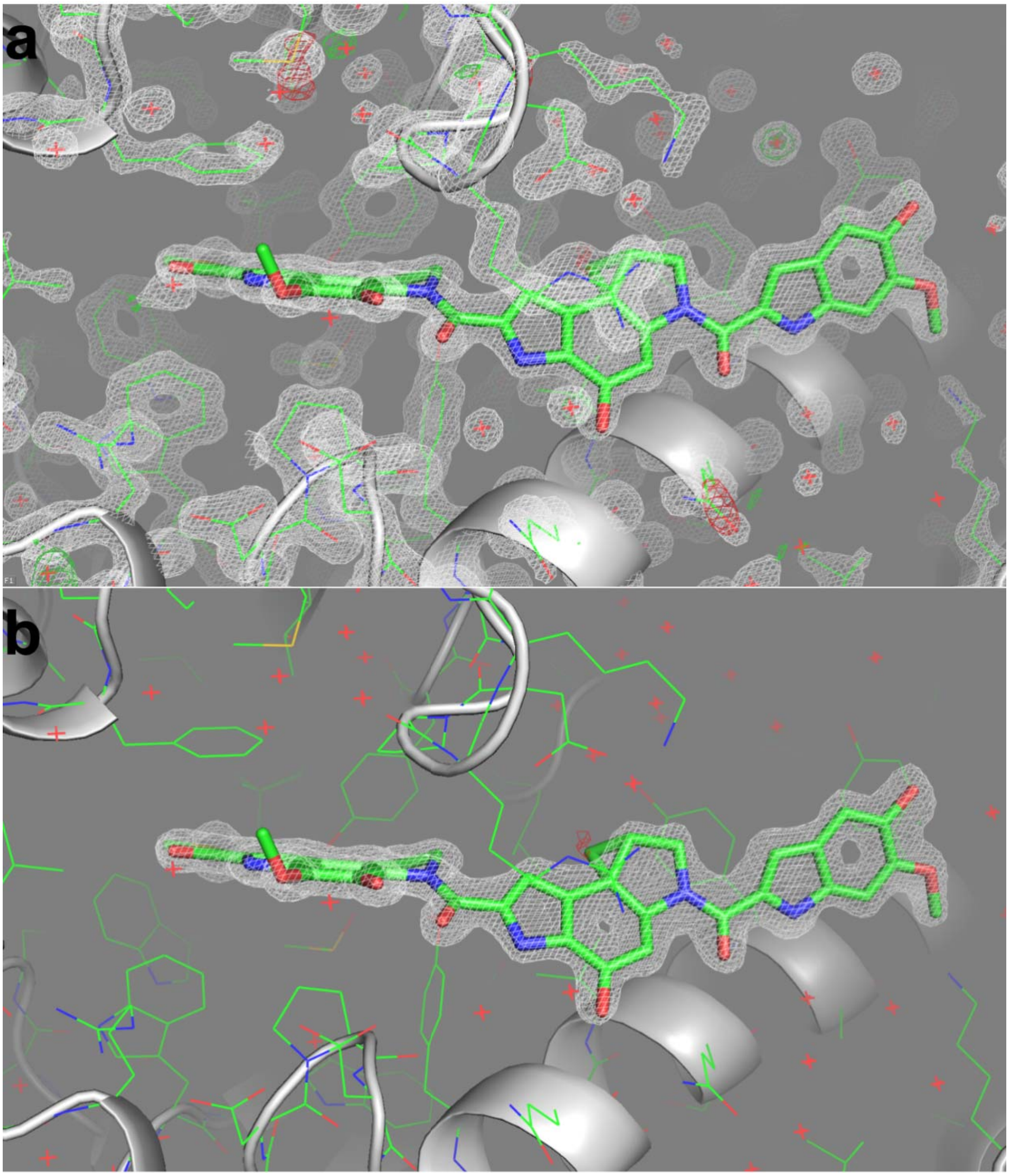

Supplementary Figure 17 | The electron density map of (a) residues (green line) around the catalytic pocket and (b) substrate YTM (green stick) in the complex structure.

White grid, 2Fo-Fc at 1.5 sigma level; green grid, Fo-Fc at 4.0 sigma level; red grid, Fo-Fc at -4.0 sigma level.

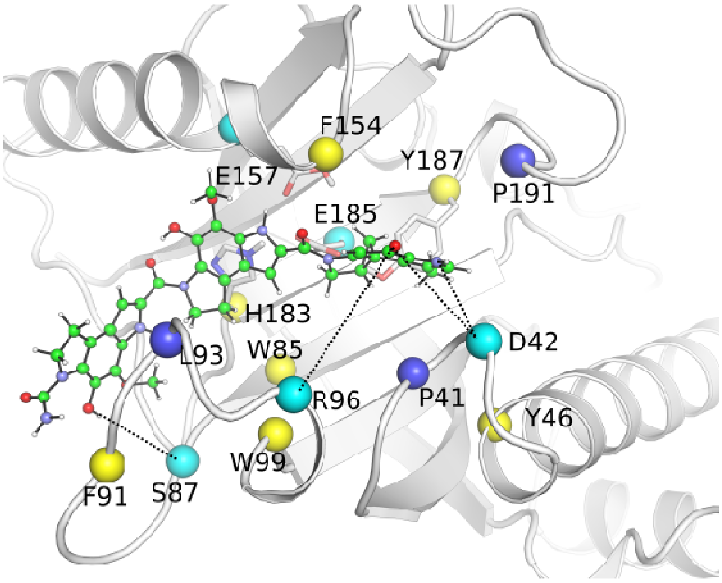

Supplementary Figure 18 | CC-1065 could be well docked in the same aromatic cage as YTM in lin2189.

Cyan spheres, polar residues; yellow spheres, aromatic residues; blue sphere, hydrophobic residues; and black dash, H-bond interactions.

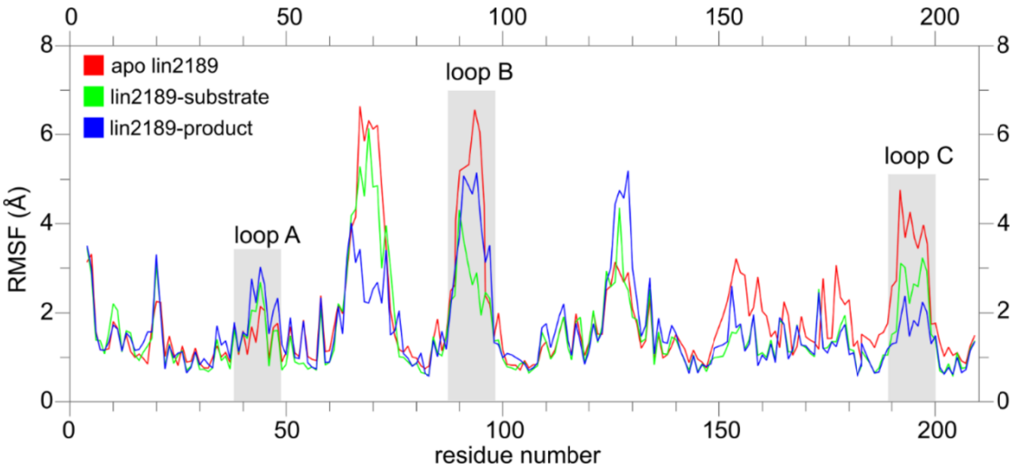

Supplementary Figure 19 | The flexibility motif of lin2189.

PCA analysis showed that loop A, loop B and loop C next to the catalytic site are very flexible. The RMSF of each amino acid for apo lin2189 (red), lin2189-YTM (green) and lin2189-**5** (blue).

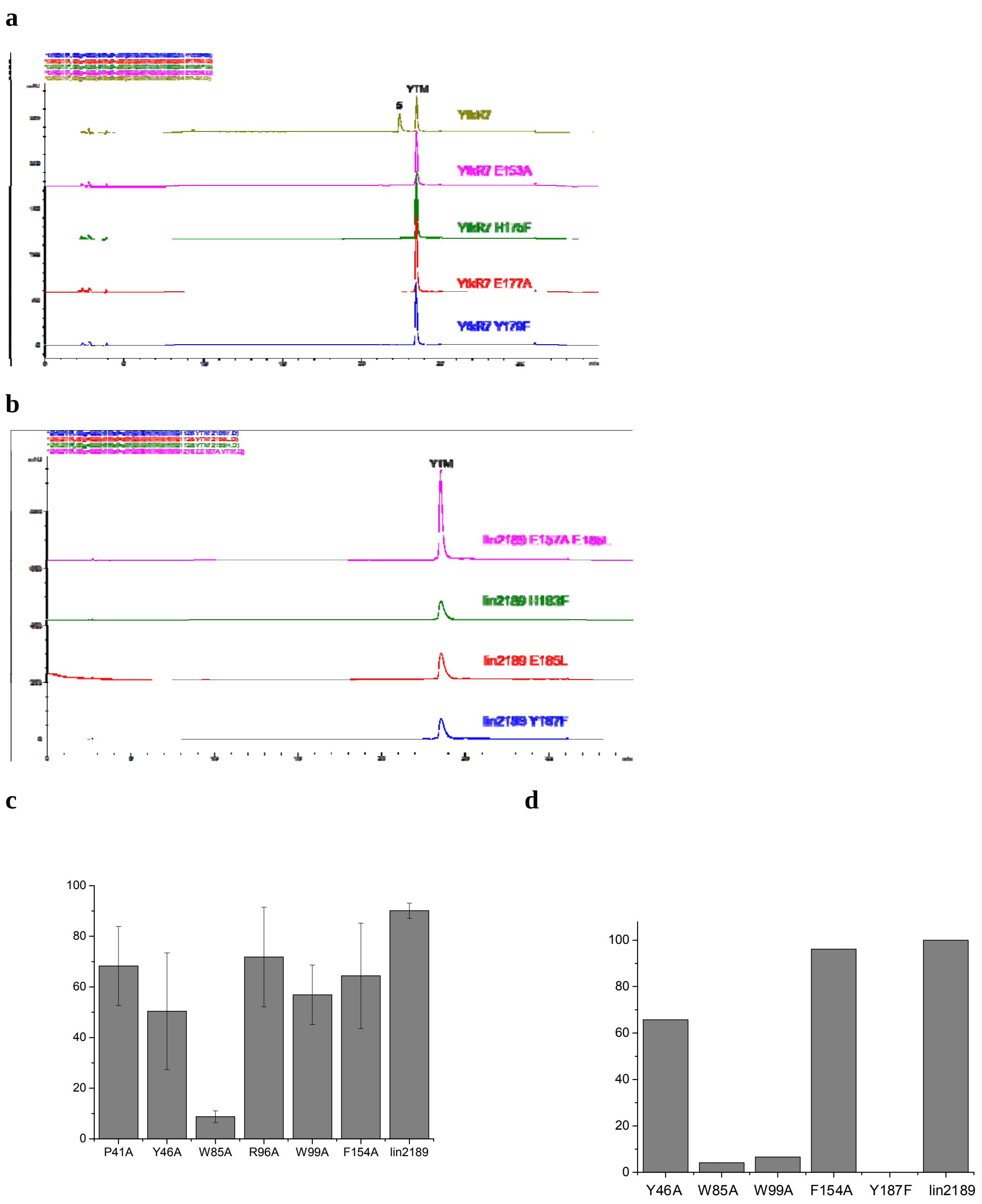

Supplementary Figure 20 | Site-directed mutagenesis experiments of CCHs.

(a) Mutagenesis of the catalytic residues of YtkR7. YtkR7 E153, H175, E177 and Y179 corresponds to the residues of lin2189 E157, H183, E185 and Y187, respectively. H175 and Y179 are believed to stabilize E177. Each enzymatic assay was performed at least two independent times. The catalytic acidic residues E157 and E185 are essential for the catalysis. (b) Mutagenesis of the catalytic residues of lin2189. H183 and Y187 are believed to stabilize E185. (c) The W85 residue of the aromatic cage has a very significant effect on YTM hydrolysis. The Y axis indicates the relative capacity of **5** production. The enzymatic assays were performed twice, and the standard error is indicated by an error bar. (d) The aromatic cage of lin2189 also has critical effects on the hydrolysis of CC-1065. The Y axis indicates the relative capacity of **7** production.

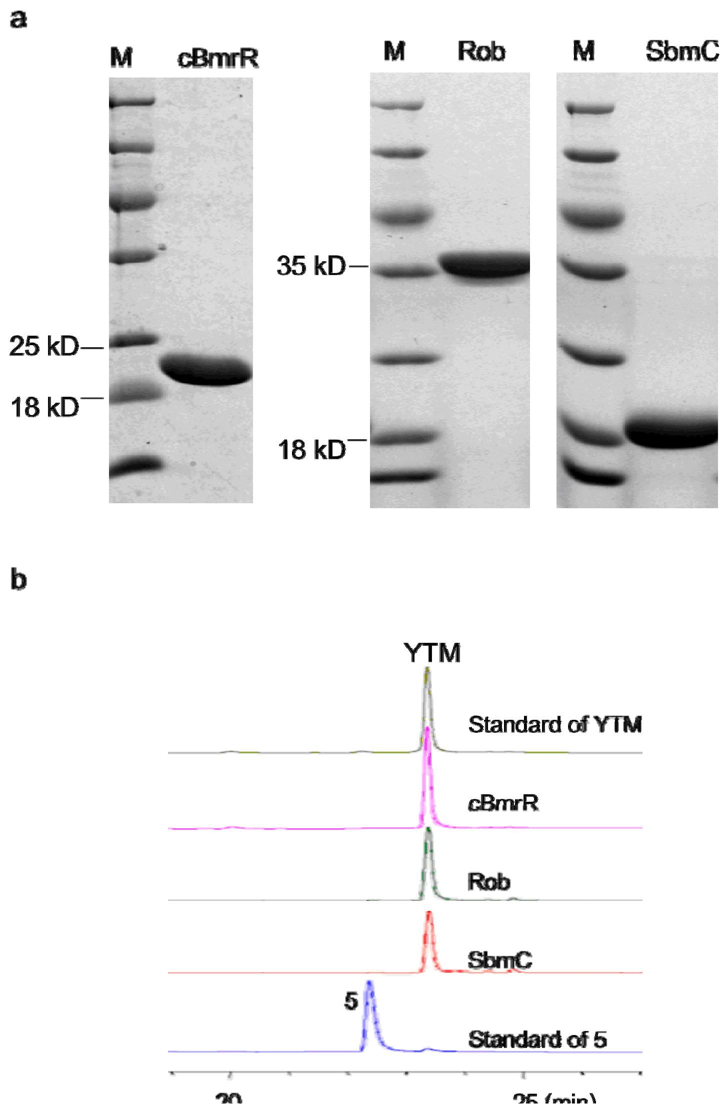

Supplementary Figure 21 | Distantly-related GyrI-like proteins could not catalyze the YTM hydrolysis.

These include cBmrR, Rob, and SbmC. cBmrR indicates a truncated form of the C-terminal GyrI-like domain of BmrR (residues 121-278). HPLC analysis with UV detection at 383 nm.

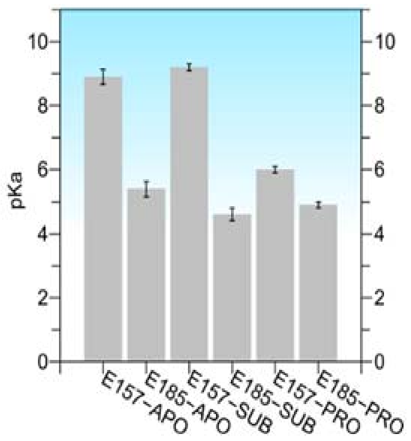

Supplementary Figure 22 | The calculated pKa values of E157 and E185 for apo-lin2189, lin2189-YTM and lin2189-5.

The standard error is indicated by an error bar.

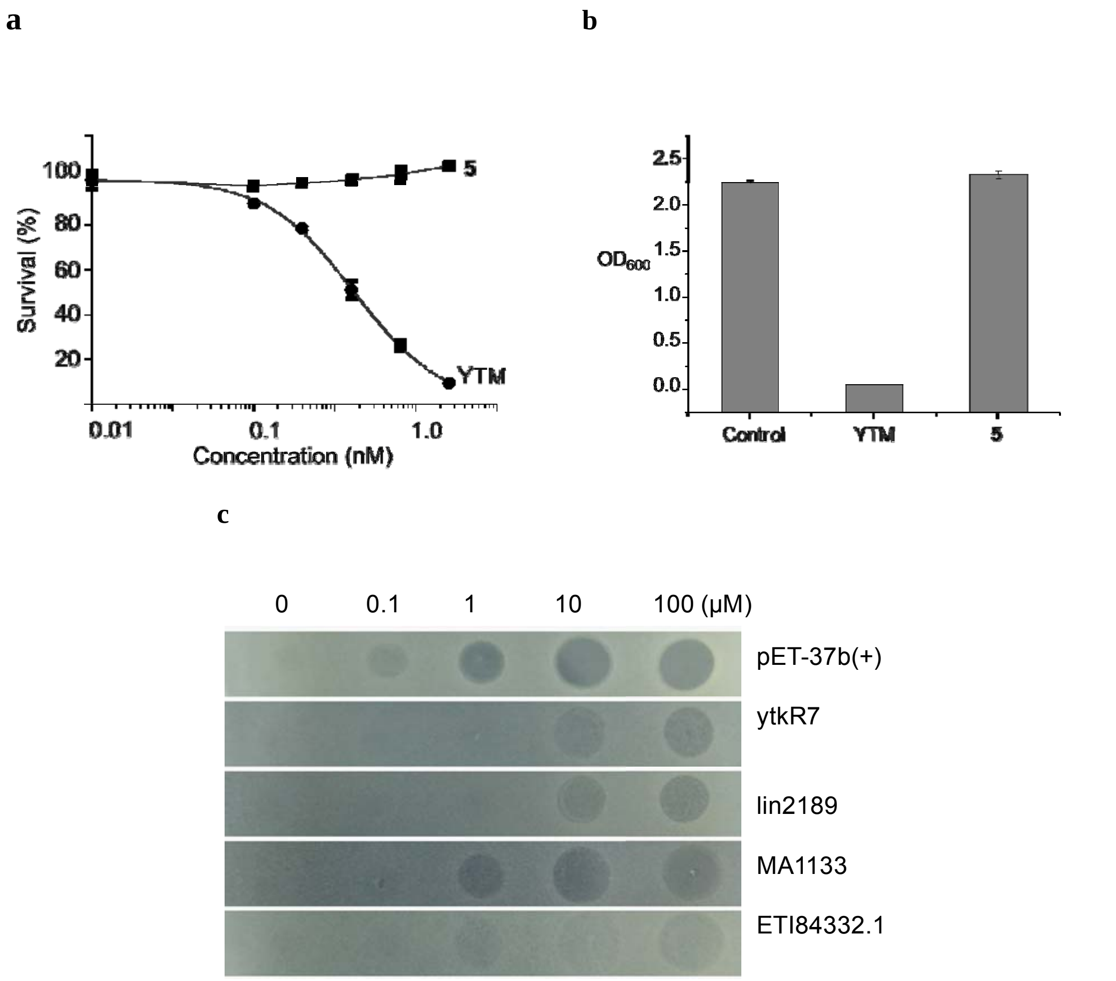

Supplementary Figure 23 | *In vitro* cytotoxicity and bacterial growth inhibition assays for YTM.

(a) *In vitro* cytotoxicity assay of Jurkat cells with the compound **5**. The IC_{50} for YTM and **5** is 0.41±0.018 and >10, 000 nM, respectively. (b) *In vitro* cytotoxicity assay of *E. coli* BL21(DE3) with the compound **5**. (c) CCHs endow bacteria with resistance to YTM.

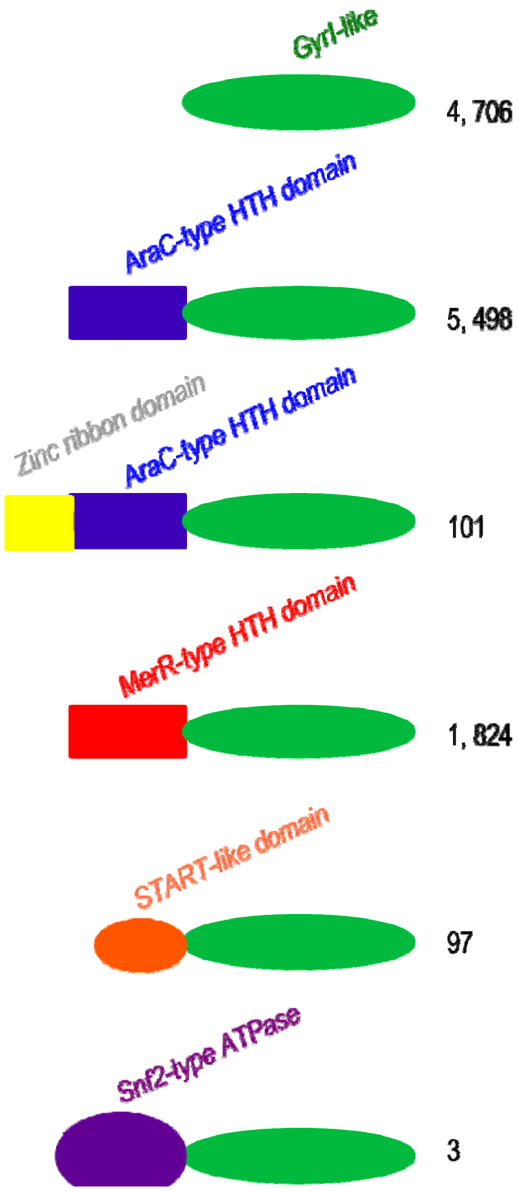

Supplementary Figure 24 | Domain organization analysis of the 12, 304 GyrI-like proteins. The 12, 304 GyrI-like proteins were retrieved from the InterPro protein database (September 2016). The domain organizations with more than 20 sequences are shown. The three Snf2-type ATPase domain containing members are also included, that is, XP_011794369.1 from *Colobus angolensis* palliatus, XP_011841664.1 from *Mandrillus leucophaeus*, and XP_014986047.1 from *Macaca mulatta*.

Antibiotics	YtkR7	Rob	SbmC
Rhodamine 6G (R6G)	2.430E-5	3.112E-5	1.692E-5
Ampicillin			
Spectinomycin			
Gentamycin			
Streptomycin			
Apramycin (Am)	3.097E-5		
Kanamycin			
Lincomycin			
Chloramphenicol			
Tetracycline (TET)	1.296E-5	9.221E-6	
Erythromycin			
Rifamycin S (RfS)	5.149E-6	5.799E-6	9.267E-6
Mitomycin C			
Vancomycin (Van)			8.445E-6
Bleomycin (Blm)	9.184E-5	>2.008E-4	
Thiostrepton			
Yatakemycin (YTM)	2.601E-5	>1.687E-5	1.177E-5
The compound **5**			

a

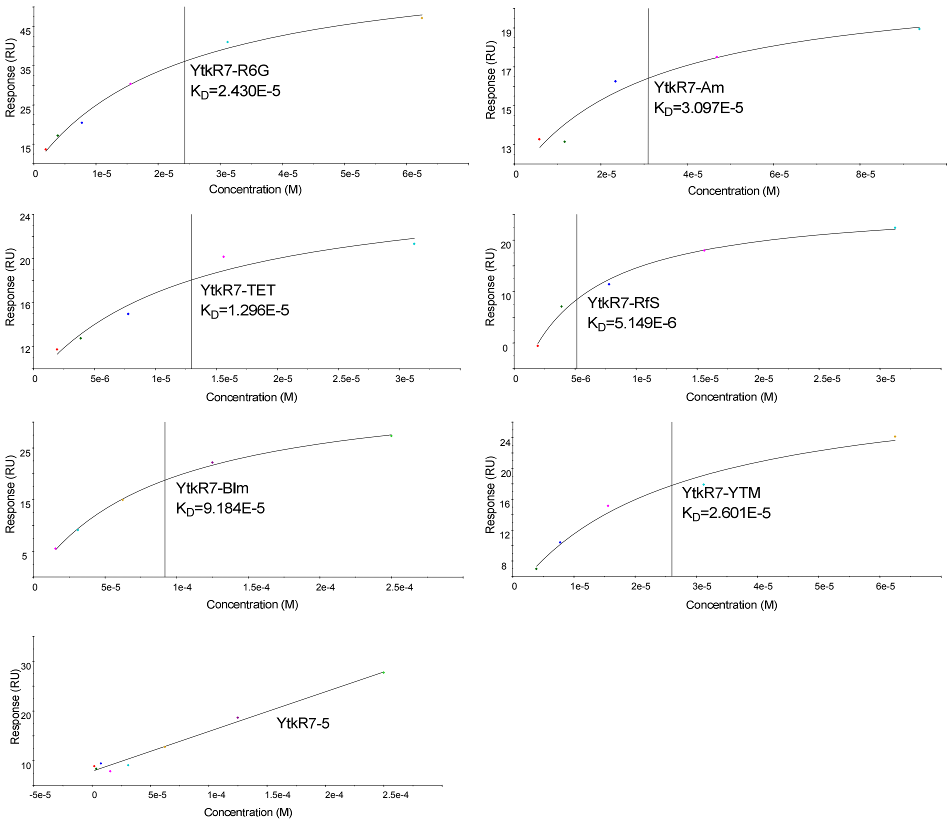

b

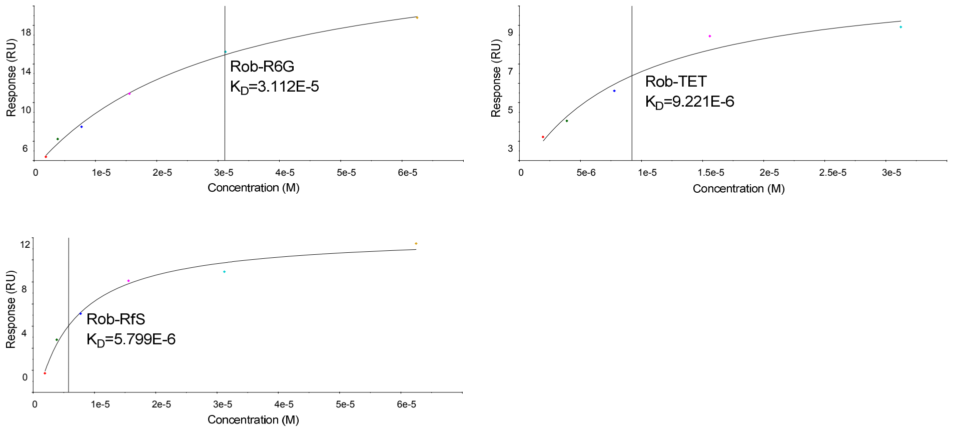

c

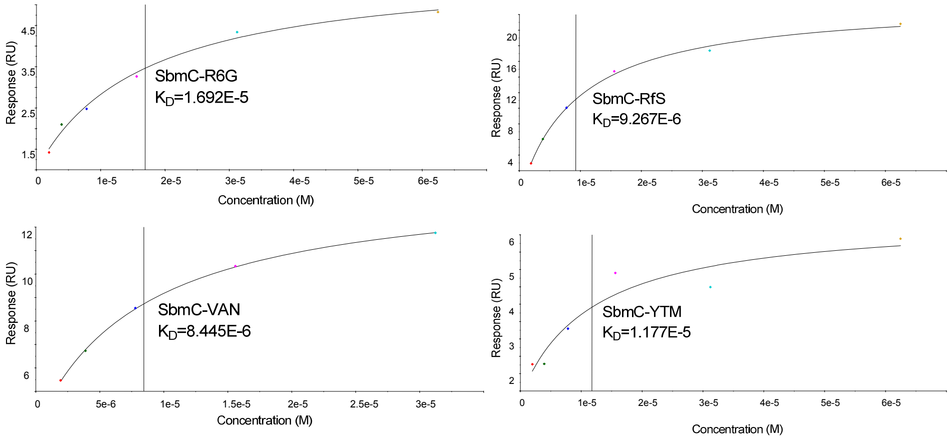

Supplementary Figure 25 | SPR analysis of the binding abilities of GyrI-like proteins to antibiotics.

The reliable dissociation constants (K_D) are indicated in the corresponding blanks, while the remaining blanks indicate that we did not observe any intermolecular interactions or that we could not get a reliable K_D value. (a), (b) and (c) indicate the binding curves for YtkR7, Rob and SbmC, respectively. Although Rob and SbmC have been reported to be involved in cellular self-protection against toxic chemicals[5,6], their ability to bind small molecules is not as yet confirmed. Here, our SPR data revealed that both proteins still retain the ability to bind promiscuous antibiotics.

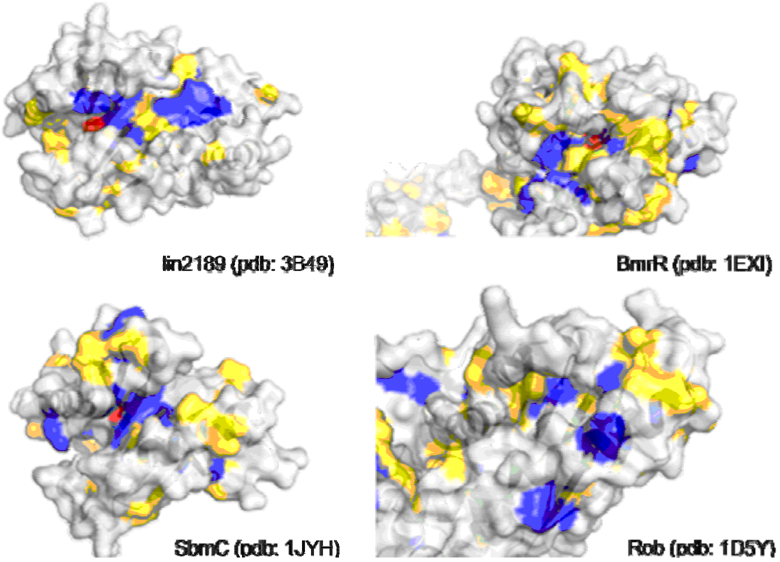

Supplementary Figure 26 | The surface maps for lin2189, BmrR, SbmC, and Rob.
The blue surface belongs to aromatic residues, while the yellow one is for hydrophobic residues. The highly conserved E185 (numbered for lin2189) of GyrI-like proteins is colored as red sticks.

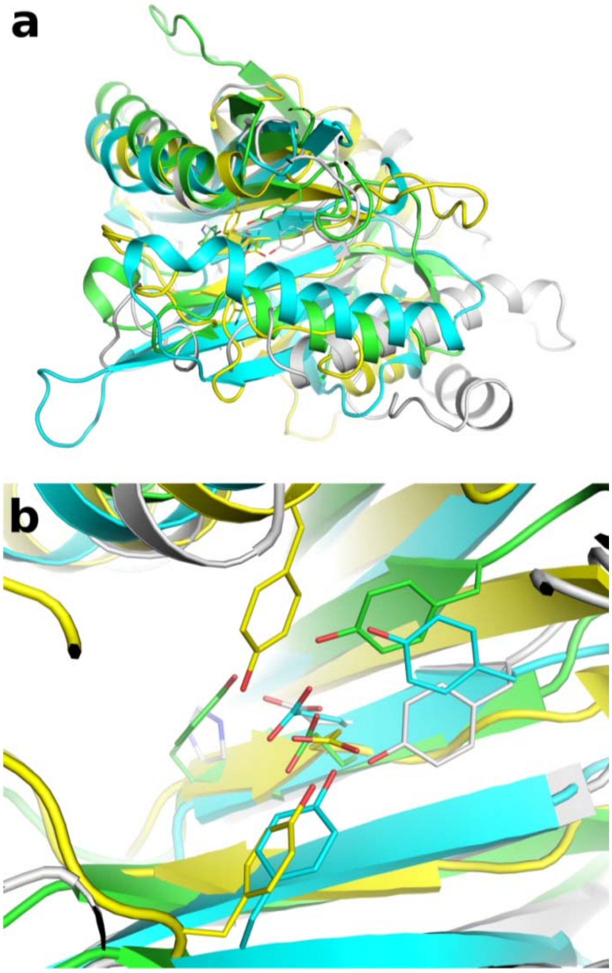

Supplementary Figure 27 | Structural superposition of representative GyrI-like proteins. The catalytic traits (including the highly conserved E185 (lin2189 number)) are indicated by sticks. Gray, lin2189 (pdb: 3B49); yellow, BmrR (1EXI); green, SbmC (1JYH); cyan, Rob (1D5Y).

References

1. Igarashi, Y. *et al.* Yatakemycin, a novel antifungal antibiotic produced by *Streptomyces* sp. TP-A0356. *J. Antibiot. (Tokyo)* **56**, 107-113 (2003).

2. Hanka, L. J., Dietz, A., Gerpheide, S. A., Kuentzel, S. L. & Martin, D. G. CC-1065 (NSC-298223), a new antitumor antibiotic. Production, *in vitro* biological activity, microbiological assays and taxonomy of the producing microorganism. *J. Antibiot. (Tokyo)* **31**, 1211-1217 (1978).

3. Gust, B., Challis, G. L., Fowler, K., Kieser, T. & Chater, K. F. PCR-targeted *Streptomyces* gene replacement identifies a protein domain needed for biosynthesis of the sesquiterpene soil odor geosmin. *Proc. Natl. Acad. Sci. USA* **100**, 1541-1546 (2003).

4. Kieser, T. *et al. Practical Streptomyces Genetics* (*John Innes Foundation,* 2000).

5. Kwon, H. J., Bennik, M. H., Demple, B. & Ellenberger, T. Crystal structure of the *Escherichia coli* Rob transcription factor in complex with DNA. *Nat. Struct. Biol.* **7**, 424-430 (2000).

6. Romanowski, M. J., Gibney, S. A. & Burley, S. K. Crystal structure of the *Escherichia coli* SbmC protein that protects cells from the DNA replication inhibitor microcin B17. *Proteins* **47**, 403-407 (2002).
