# Supplementary material for: GyrI-like proteins catalyze cyclopropanoid hydrolysis to confer cellular protection
Source: Nat Commun. 2017 Nov 14;8:1485. doi: 10.1038/s41467-017-01508-1 (PMC5684135; doi:10.1038/s41467-017-01508-1)
Supplement: Supplementary file 2 — Description of Additional Supplementary Files [file 41467_2017_1508_MOESM2_ESM.docx]

**Descriptions of Additional Supplementary Files:**

File Name: Supplementary Movie 1

Description:  The intrinsic mobility of lin2189 calculated from the principal component analysis. The cyan vector length correlates with the domain-motion scale. Yellow ball-and-stick, the substrate molecule (YTM).

File Name: Supplementary Movie 2

Description: The substrate YTM (Yellow ball-and-stick) entrance pathway sampled by metaMD simulations. Blue, loop A; green, loop B; and pink, loop C.

File Name: Supplementary Movie 3

Description: The product 5 (Yellow ball-and-stick) leaving pathway sampled by metaMD simulations. Blue, loop A; green, loop B; and pink, loop C.
